# Functional and genetic analysis of viral receptor ACE2 orthologs reveals a broad potential host range of SARS-CoV-2

**DOI:** 10.1073/pnas.2025373118

**Published:** 2021-03-03

**Authors:** Yinghui Liu, Gaowei Hu, Yuyan Wang, Wenlin Ren, Xiaomin Zhao, Fansen Ji, Yunkai Zhu, Fei Feng, Mingli Gong, Xiaohui Ju, Yuanfei Zhu, Xia Cai, Jun Lan, Jianying Guo, Min Xie, Lin Dong, Zihui Zhu, Jie Na, Jianping Wu, Xun Lan, Youhua Xie, Xinquan Wang, Zhenghong Yuan, Rong Zhang, Qiang Ding

**Affiliations:** ^a^Center for Infectious Disease Research, School of Medicine, Tsinghua University, 100084 Beijing, China;; ^b^Key Laboratory of Medical Molecular Virology, Biosafety Level 3 Laboratory, School of Basic Medical Sciences, Shanghai Medical College, Fudan University, Shanghai 200032, China;; ^c^School of Life Sciences, Tsinghua University, 100084 Beijing, China;; ^d^Key Laboratory of Structural Biology of Zhejiang Province, School of Life Sciences, Westlake University, 310024 Hangzhou, China;; ^e^Institute of Biology, Westlake Institute for Advanced Study, 310024 Hangzhou, China;; ^f^Beijing Advanced Innovation Center for Structural Biology, Tsinghua University, 100084 Beijing, China

**Keywords:** COVID-19, SARS-CoV-2, ACE2, host range, intermediate host

## Abstract

The pandemic of COVID-19, caused by SARS-CoV-2, is a major global health threat. Epidemiological studies suggest that bats (*Rhinolophus affinis*) are the natural zoonotic reservoir for SARS-CoV-2. However, the host range of SARS-CoV-2 and intermediate hosts that facilitate its transmission to humans remain unknown. The interaction of coronavirus with its host receptor is a key genetic determinant of host range and cross-species transmission. SARS-CoV-2 uses angiotensin-converting enzyme 2 (ACE2) as the receptor to enter host cells in a species-dependent manner. In this study, we characterized the ability of ACE2 from diverse species to support viral entry. By analyzing the conservation of five residues in two virus-binding hotspots of ACE2 (hotspot 31Lys and hotspot 353Lys), we predicted 80 ACE2 proteins from mammals that could potentially mediate SARS-CoV-2 entry. We chose 48 ACE2 orthologs among them for functional analysis, and showed that 44 of these orthologs—including domestic animals, pets, livestock, and animals commonly found in zoos and aquaria—could bind the SARS-CoV-2 spike protein and support viral entry. In contrast, New World monkey ACE2 orthologs could not bind the SARS-CoV-2 spike protein and support viral entry. We further identified the genetic determinant of New World monkey ACE2 that restricts viral entry using genetic and functional analyses. These findings highlight a potentially broad host tropism of SARS-CoV-2 and suggest that SARS-CoV-2 might be distributed much more widely than previously recognized, underscoring the necessity to monitor susceptible hosts to prevent future outbreaks.

## Significance

COVID-19, caused by SARS-CoV-2, is a major global health threat. The host range of SARS-CoV-2 and intermediate hosts that facilitate its transmission to humans remain unknown. We found that SARS-CoV-2 has the potential to infect a broad range of mammalian hosts, including domestic animals, pets, livestock, and animals commonly found in zoos and aquaria. Those species may be at risk for human-to-animal or animal-to-animal transmissions of SARS-CoV-2. Our study highlights the importance of banning illegal wildlife trade and consumption, and enforcing the importance of surveilling animals in close contact with humans as potential zoonotic reservoirs to prevent outbreaks in the future.

Coronaviruses are a group of positive-stranded, enveloped RNA viruses that circulate broadly among humans, other mammals, and birds, causing respiratory, enteric, or hepatic diseases (1). In the last two decades, coronaviruses have caused three major outbreaks: severe acute respiratory syndrome (SARS), Middle East respiratory syndrome (MERS), and the recent coronavirus disease 2019 (COVID-19) (2, 3). As of December 7, 2020, COVID-19 has already caused 50 million infections, leading to 1 million deaths globally. The pathogen responsible is a novel coronavirus-severe acute respiratory syndrome coronavirus 2 (SARS-CoV-2) (4, 5). Phylogenetic and epidemiological analyses suggest that SARS-CoV, MERS-CoV, and SARS-CoV-2 likely originated from bats, with SARS-CoV spreading from bats to palm civets to humans, and MERS-CoV spreading from bats to camel to humans (6). However, the intermediate host of SARS-CoV-2, fueling spillover to humans, remains unknown.

The SARS-CoV-2 genome encodes a spike (S) protein, the receptor-binding domain (RBD) of which binds the cellular receptor angiotensin-converting enzyme 2 (ACE2) to mediate viral entry (5, 7). Following binding of ACE2, the S protein is subsequently cleaved by the host transmembrane serine protease 2 (TMPRSS2) to release the spike fusion peptide, promoting virus entry into target cells (7). It has been demonstrated that the interaction of a virus with species-specific receptors is a primary determinant of host tropism and therefore constitutes a major interspecies barrier at the level of viral entry (8). For example, murine ACE2 does not efficiently bind the SARS-CoV or SARS-CoV-2 S protein, hindering viral entry into murine cells; consequently, a human ACE2 transgenic mouse was developed as an in vivo model to study the infection and pathogenesis of these two viruses (9, 10).

ACE2 is expressed in a diverse range of species throughout the subphylum *Vertebrata*. Several recent studies demonstrated that ferrets, cats, dogs, and some nonhuman primates are susceptible to SARS-CoV-2 (11–15). However, the exact host tropism of SARS-CoV-2 remains unknown and it is urgent to identify the putative zoonotic reservoirs to prevent future outbreaks. Numerous studies have predicted ACE2 orthologs/SARS-CoV-2 S binding affinity or energies but lack of support by virus infection experimentation (16–21). In this study, we experimentally assessed ACE2 orthologs from a broad range of species for their ability to support SARS-CoV-2 entry. Our data demonstrate that an evolutionarily diverse set of ACE2 species variants can mediate SARS-CoV-2 entry, suggesting that SARS-CoV-2 has a broad host range at the level of virus entry that may contribute to cross-species transmission and viral evolution.

## Results

### Evolutionary and Phylogenetic Analyses of ACE2 Orthologs from a Diversity of Species.

**Fig. 1.**
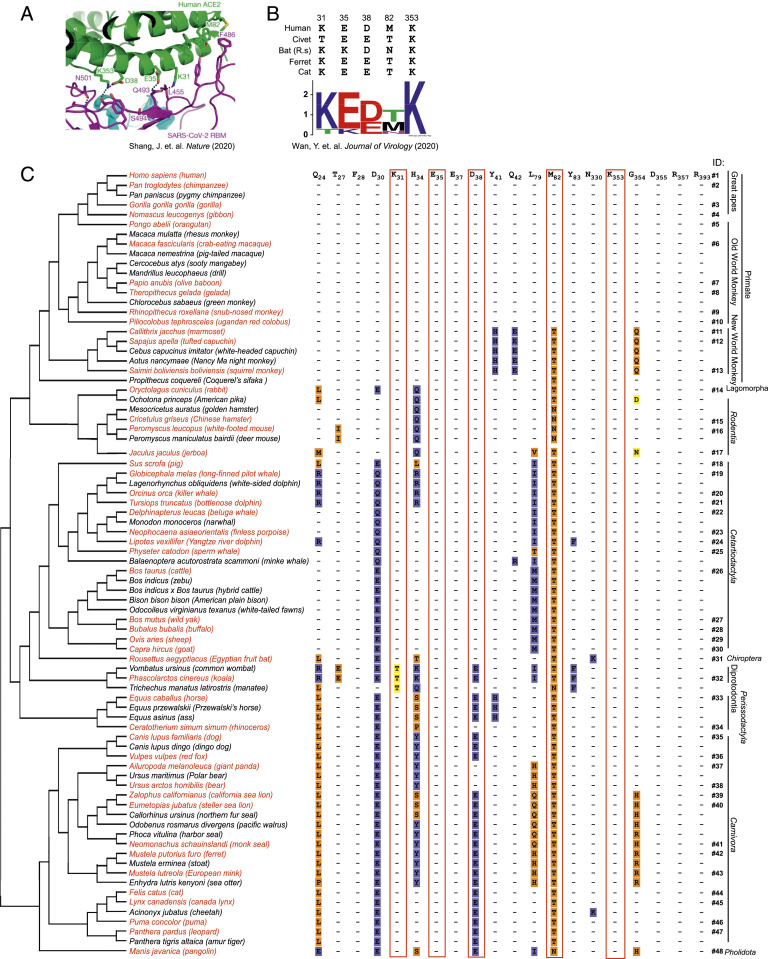
Phylogenetic analysis of ACE2 orthologs with potential to support SARS-CoV-2 entry and alignment of ACE2 residues at the interface with the viral S protein (12). (*A*) The interface between the SARS-CoV-2 receptor binding motif (RBM) and human ACE2 (PDB: 6VW1) (22). The hotspot 31K consists of a salt bridge between 31K and 35E, and hotspot 353K consists of a salt bridge between 353K and 38D. 486F of the SARS-CoV-2 RBM inserts into a hydrophobic pocket of M82 of ACE2 to further stabilize hotspot 31K. (*B*) Critical changes in virus-contacting residues of K31 and K353 hotspots in ACE2 from different host species susceptible to SARS-CoV-2 infection. The sequence logo was generated using WebLogo (weblogo.berkeley.edu/). GenBank accession numbers for ACE2 are as follows: NM_001371415.1 (human), AAX63775.1 (civet), KC881004.1 (bat), AB208708 (ferret), NM_001039456 (cat) (23). (*C*) The sequences of 80 ACE2 proteins (Dataset S1) were analyzed and the phylogenetic tree was built. The residues of human ACE2 at the SARS-CoV-2 RBD/ACE2 interface are shown. The five residues constituting 31K and 353K hotspots are highlighted in a red box. The ID number of each species in subsequent experiments is labeled on the right. Only the residues different from human are shown and each amino acid substitution is colored according to its classification as nonconservative (orange), semiconservative (yellow), or conservative (blue), as compared to the human residue. The species highlighted in red were chosen for further functional analysis.

ACE2, a peptidase expressed at the surface of lung epithelial cells and other tissues that regulate the renin-angiotensin-aldosterone system, is the cellular receptor for SARS-CoV-2 (5). We analyzed the protein sequences of 295 ACE2 orthologs recorded in the National Center for Biotechnology Information (NCBI) database, from birds (75 species), alligators (4 species), turtles (4 species), lizards (9 species), mammals (130 species), amphibians (4 species), coelacanths (1 species), bone fish (67 species), and cartilaginous fish (1 species) (*SI Appendix*, Fig. S1). As reported previously, there are two hotspots in ACE2 at the SARS-CoV-2 RBD/human ACE2 binding interface: hotspots K31 and K353, which are constituted by five amino acids and provide a substantial amount of energy to the virus-receptor binding interactions and regarded as important determinants of host range (Fig. 1 *A* and *B*) (22, 23). Based on the conservation of these five critical residues in known susceptible species (31K/T, 35E/K, 38D/E, 82T/M/N, and 353K) as reported by Li and colleagues (22, 23), we carried out primary sequence alignment across the 295 ACE2 proteins (*Materials and Methods* and *SI Appendix*, Fig. S1). Our analysis revealed that 80 ACE2 orthologs from 295 species exactly contained the relevant amino acids at positions 31, 35, 38, 82, and 353, which we deduce would allow them to function as SARS-CoV-2 receptors (Fig. 1 *A* and *B* and *SI Appendix*, Fig. S1). All of the 80 ACE2 orthologs were derived from mammals with protein size ranging from 790 to 811 amino acids (Dataset S1). Fifty other ACE2 orthologs from mammals are summarized in Dataset S2. Consistently, species in Dataset S2, such as mouse, Chinese tree shrew, and guinea pigs were not susceptible to SARS-CoV-2 infection, as demonstrated by other studies (24, 25).

Next, we performed phylogenetic analysis of these 80 ACE2 orthologs to explore their potential function in mediating virus infection and to gain insights into the evolution of the ACE2 protein (Fig. 1 *C*, *Left*). Additionally, we aligned the 20 residues of ACE2 located at the interface with the SARS-CoV-2 S protein (22, 26–28) (Fig. 1 *C*, *Right*). Of note, the 20 residues at the ACE2–S protein interface were identical across the Catarrhini, which includes great apes and Old World monkeys. However, these residues in the ACE2 orthologs of New World monkeys were less conserved. For example, Y41 and Q42 in human ACE2 are responsible for the formation of hydrogen bonds with S protein and are highly conserved across all other species but are substituted by H and E, respectively, in New World monkeys. In nonprimate mammals, an increasing number of substitutions are evident, even in residues such as Q24, D30, D38, and Y83 that form hydrogen bonds or salt-bridges with the S protein (Fig. 1*C*).

### Interaction of ACE2 Proteins with SARS-CoV-2 S Protein.

Among those 80 orthologs with potential viral receptor activity, we chose 48 representative ACE2 orthologs for further functional analysis according to following criteria: Species are frequently in close contact with human including pets and livestock; species are model animals used in biomedical research, such as marmosets and crab-eating macaques; endangered species, such as giant pandas, golden snub-nosed monkeys; and some wild animals (Fig. 1*C* and *SI Appendix*, Fig. S1). We assessed whether they support SARS-CoV-2 entry by ectopically expressing each ortholog in A549 cells, which derive from human lung carcinoma with limited endogenous ACE2 expression (7, 29). Besides the 48 ACE2 orthologs above, we also included mouse ACE2 as the negative control (5).

**Fig. 2.**
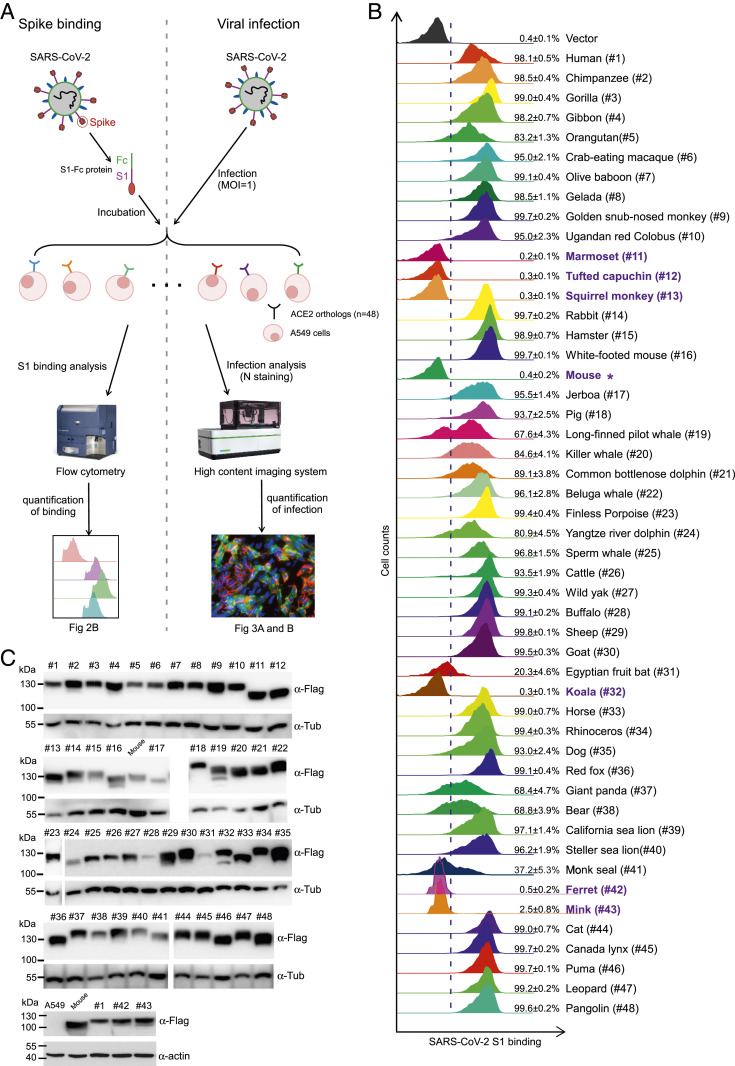
Binding of the SARS-CoV-2 spike protein to different ACE2 orthologs. (*A*) Schematic of testing the efficiency of ACE2 orthologs binding with viral spike and the abilities to mediate virus entry. (*B*) A549 cells were transduced with ACE2 orthologs of the indicated species, incubated with the recombinant S1 domain of SARS-CoV-2 S protein C-terminally fused with Fc, and then stained with goat anti-human IgG (H + L) conjugated to Alexa Fluor 647 for flow cytometry analysis. Binding efficiencies are expressed as the percent of cells positive for S1-Fc among the ACE2 expressing cells from one representative experiment with three replicates. This experiment was independently repeated three times with similar results. The ACE2 orthologs exhibited binding efficiency with S1-Fc < 5% are highlighted in purple. (*C*) Western blotting of cell lysates of A549 cells transduced with lentiviruses expressing FLAG-tagged ACE2 orthologs. The cell lysates were probed with Flag antibody, followed by incubating with horseradish peroxidase (HRP)-conjugated secondary antibody and developed using SuperSignal West Pico chemiluminescent substrate and the signals were detected using the Luminescent image analyzer (GE Healthcare). Tubulin or actin served as the loading control.

Binding of the SARS-CoV-2 S protein to ACE2 is a prerequisite for viral entry. To examine this, we employed a cell-based assay that used flow cytometry to assess the binding of the S protein to different ACE2 orthologs (Fig. 2 *A* and *B*). We cloned the codon-optimized cDNA of ACE2 orthologs, each with a C-terminal FLAG tag, into a bicistronic lentiviral vector (pLVX-IRES-zsGreen1) that expresses the fluorescent protein zsGreen1 via an internal ribosome entry site (IRES) element and can be used to monitor transduction efficiency. To avoid the excess of the SARS-CoV-2 S protein, leading to saturation of the interaction of the S protein with ACE2 receptors on target cells, we performed a titration assay to determine the concentration of S1-Fc (a purified fusion protein consisting of the S1 domain of SARS-CoV-2 S protein and an Fc domain of human lgG) used for quantification of binding efficiency as 1 μg/mL (*SI Appendix*, Fig. S2). Next, S1-Fc was incubated with A549 cells transduced with the ACE2 orthologs. Binding of S1-Fc to ACE2 was then quantified by flow cytometry as the percent of cells positive for S1-Fc among the ACE2-expressing cells (*SI Appendix*, Fig. S3). As expected, the binding of S1-Fc to A549 cells expressing mouse ACE2 was very low and comparable to that of the empty vector control, whereas S1-Fc protein efficiently bound to A549 cells expressing human ACE2, which is consistent with previous reports (5). We found that ACE2 from 42 of 48 species could bind the S1-Fc protein, albeit with various efficiencies (20 to 100%) (Fig. 2*B*).

In contrast, ACE2 from *Callithrix jacchus* (marmoset, #11), *Sapajus apella* (tufted capuchin, #12), *Saimiri boliviensis boliviensis* (squirrel monkey, #13), all New World monkeys, and *Phascolarctos cinereus* (koala, #32) failed to bind S1-Fc (Fig. 2*B*). Our findings are consistent with the recent report that experimental SARS-CoV-2 infection could be established in Old World monkeys (*Macaca mulatta* and *Macaca fascicularis*; #6 in our analysis) but not in New World monkeys (*C. jacchus*, marmoset; #11 in our analysis) (12). Interestingly, ferret and mink are susceptible to SARS-CoV-2 infection (11, 30); however, their ACE2 orthologs exhibited negligible activities to bind with S1-Fc (Fig. 2*B*, #42 and #43). Surface plasmon resonance, a quantitative and sensitive technique for kinetic studies of biomolecular interactions, demonstrated that the ferret or mink ACE2 could bind SARS-CoV-2 spike, albeit with limited affinity; in contrast, in interaction of mouse or rat ACE2 with spike protein still cannot be detected (*SI Appendix*, Fig. S3*B*).

The limited or undetectable interaction of certain ACE2 orthologs with the S1-Fc protein was not due to low expression of ACE2 or alteration of its cell surface localization. The expression of ACE2 orthologs in A549 cells following transduction was assessed by Western blot using an anti-FLAG antibody. ACE2 proteins were readily detected at the expected size of 100 to 130 kDa in all of the ACE2 ortholog-transduced A549 cells (Fig. 2*C*). The differences in molecular weights are likely attributable to their varying degrees of glycosylation. To confirm the cell surface localization of ACE2 orthologs, we detected or visualized the location of a subset of ACE2 orthologs, which exhibited binding efficiency with S1-Fc less than 70% (Fig. 2*B* and *SI Appendix*, Fig. S4) by cell surface flow cytometry or confocal fluorescence microscopy. The polyclonal ACE2 antibody could cross-react with most of the ACE2 orthologs (except long-finned pilot whale and koala ACE2s) for detection of cell surface ACE2 by flow cytometry (*SI Appendix*, Fig. S4*A*). The long-finned pilot whale and koala ACE2 were therefore cloned as a carboxyl terminus fusion with EGFP and their localization could be visualized by confocal fluorescence microscopy (*SI Appendix*, Fig. S4 *B* and *C*).

These results showed that all the ACE2 orthologs could be expressed and localized at the cell surface with comparable levels (*SI Appendix*, Fig. S4), excluding the possibilities that the limited or undetectable binding efficiencies of ACE2 orthologs with S1-Fc was attributed by their varied cell surface localization. In parallel, we used the HeLa cells, which are also lack of endogenous ACE2 expression (5), to perform the ACE2 ortholog-S protein binding assay (*SI Appendix*, Fig. S5) and the binding results are consistent with that of A549 cells, excluding the cell-specific artifact. In summary, these results demonstrate that ACE2 proteins from a broad range of diverse species can bind to the SARS-CoV-2 S protein, suggesting that these species may indeed be capable of mediating viral uptake.

### Functional Assessment of ACE2 Orthologs in SARS-CoV-2 Entry.

**Fig. 3.**
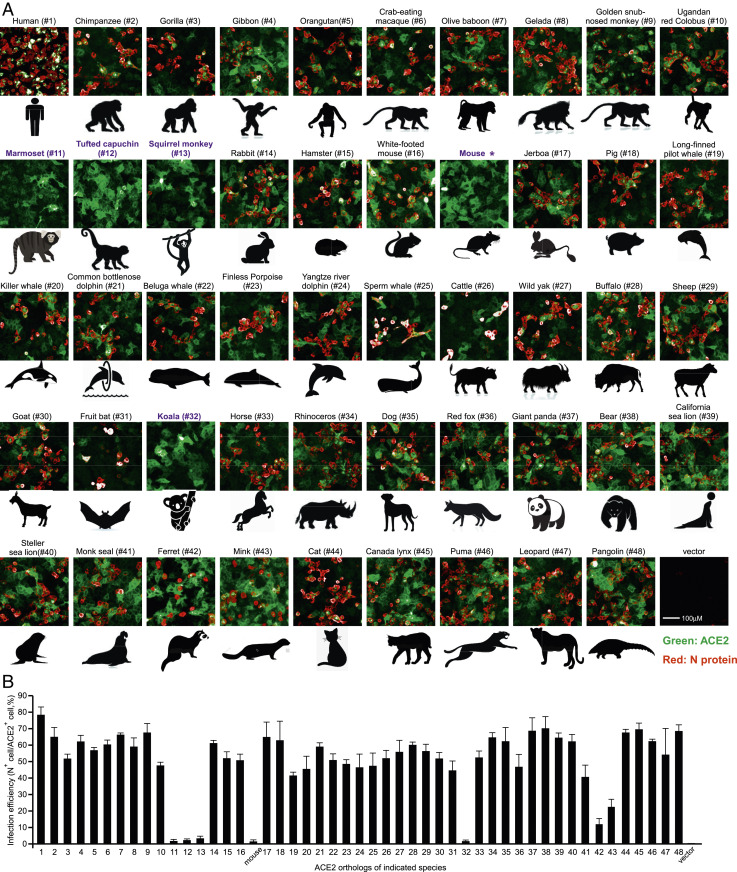
Functional assessment of ACE2 orthologs mediating SARS-CoV-2 virus entry. (*A*) A549 cells transduced with lentiviruses expressing ACE2 orthologs were infected with SARS-CoV-2 virus (MOI = 1). Expression of the viral N protein or ACE2 orthologs was visualized by Operetta High Content Imaging System (PerkinElmer). Viral N protein (red) and ACE2 ortholog (green) are shown. Marmoset (#11), tufted capuchin (#12), squirrel monkey (#13), and koala (#32) were nonpermissive to SARS-CoV-2 infection, highlighted in purple. This experiment was independently repeated three times with similar results and the representative images are shown. (*B*) The images were analyzed and quantified using PerkinElmer Harmony high-content analysis software 4.9. The infection efficiency represents the percentage of SARS-CoV-2–infected cells/ACE2^+^ cells (*y* axis). The *x* axis represents ACE2 orthologs. Error bars represent the SD of the mean from one representative experiment with three biological replicate samples and this experiment was independently repeated three times.

To test directly whether different ACE2 orthologs can indeed mediate viral entry, we performed genetic complementation experiments in A549 cells. A549 cells ectopically expressing individual ACE2 orthologs were infected with SARS-CoV-2 (multiplicity of infection [MOI] = 1). At 48 h postinfection, the complemented A549 cells were fixed and underwent immunofluorescent staining for ACE2 ortholog proteins and intracellular viral nucleocapsid (N) protein, an indicator of virus replication (Fig. 2*A*). In addition, the proportional relationship between virus quantity and N staining could be established in A549-human ACE2 cells, as evidenced that N protein-positive cells are correlated with the dose of virus infection (*SI Appendix*, Fig. S6). Therefore, the infection efficiencies (percentage of viral N^+^ cells among ACE2^+^ cells) of SARS-CoV-2 with each ACE2 ortholog-transduced A549 cells could be analyzed and quantified by the Operetta High Content Imaging System (Fig. 3). As expected, A549 cells expressing mouse ACE2 were not permissive to SARS-CoV-2 infection, while those expressing human ACE2 (#1) were permissive (Fig. 3). Intriguingly, ferret and mink ACE2, which exhibited limited binding activity with SARS-CoV-2 S1, could functionally mediate authentic virus entry with 12% or 23% infection efficiency, respectively, which are less efficient than human ACE2 (78% infection efficiency) (Fig. 3*B*).

Consistent with our binding data, A549 cells expressing ACE2 orthologs from marmoset (#11), tufted capuchin (#12), squirrel monkey (#13), or koala (#32) were nonpermissive to SARS-CoV-2 infection; the serine protease TMPRSS2 could facilitate SARS-CoV-2 entry (7, 31), and its expression was undetected in our A549 cells as previously reported (*SI Appendix*, Fig. S7*A*) (32). Similarly, the ectopic expression of TMPRSS2 only could not render the susceptibility of A549 cells to SARS-CoV-2 (*SI Appendix*, Fig. S7*B*). In line with the data in A549 cells, squirrel monkey and koala ACE2 could not support SARS-CoV-2 entry in A549 cells expressing human TMPRSS2 (*SI Appendix*, Fig. S7), which suggest that those ACE2 orthologs being unable to mediate virus entry is not due to lack of TMPRSS2. In addition, we also infected the HeLa cells transduced with ACE2 orthologs with authentic virus and the results are similar with that of A549 cells (*SI Appendix*, Fig. S8). In sum, of 48 species selected, 44 ACE2 orthologs supported the SARS-CoV-2 infection, albeit with various efficiencies compared with human ACE2 (Fig. 3 and *SI Appendix*, Fig. S8).

### The Genetic Determinants of ACE2 from New World Monkeys That Restrict SARS-CoV-2 Entry.

**Fig. 4.**
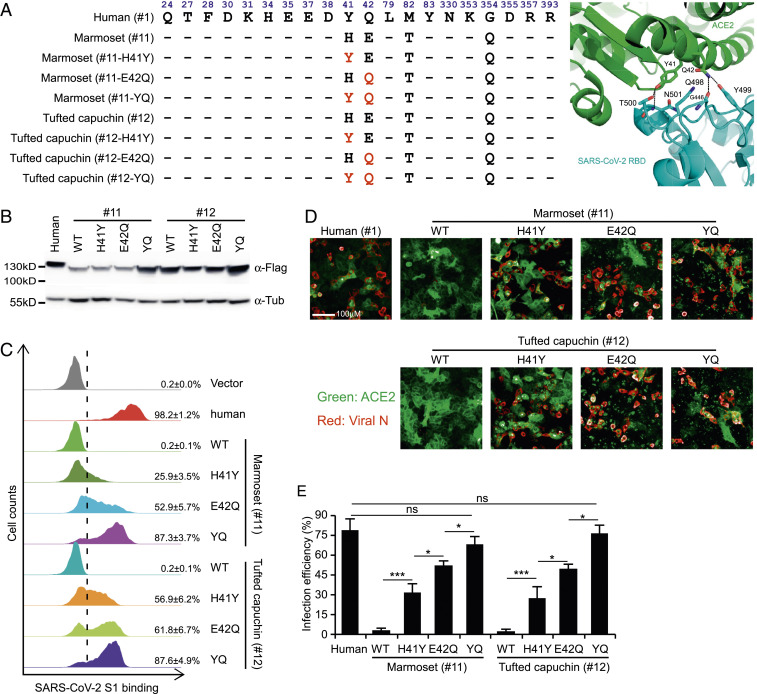
Identification of the species-specific genetic determinants of ACE2 restriction of SARS-CoV-2 entry. (*A*, *Left*) Alignment of the contacting residues of human ACE2 (distance cutoff of 4 Å) at the SARS-CoV-2 RBD/ACE2 interface with orthologs from the New World monkeys marmoset (#11) and tufted capuchin (#12). The mutations introduced into marmoset (#11) and tufted capuchin (#12) ACE2 at positions 41 and 42 are indicated in red. (*Right*) The binding interface of human ACE2 with SARS-CoV-2 RBD surrounding ACE2 Y41 and Q42. Residue Y41 forms hydrogen bonds with T500 and N501 of SARS-CoV-2 RBD, and Q42 can also interact with G446 or Y449 by hydrogen bonds. The differences in ACE2 from New World monkeys may disrupt the hydrogen-bonding interactions and impair the binding with SARS-CoV-2 spike. The PDB ID code of the complex of human ACE2 with SARS-CoV-2 is 6M0J. (*B*) The WT or humanized ACE2 orthologs expression was confirmed by immunoblotting assay. (*C*) A549 cells transduced with ACE2 variants were incubated with the S1-Fc, and then stained with goat anti-human IgG (H + L) conjugated to Alexa Fluor 647 for flow cytometry analysis. Binding efficiencies are expressed as the percent of cells positive for S1-Fc among the ACE2-expressing cells. Error bars represent the SD of the mean from one representative experiment with three biological replicate samples. This experiment was independently repeated three times with similar results. (*D* and *E*) A549 cells transduced with ACE2 orthologs of the indicated species or mutants were infected with SARS-CoV-2 virus (MOI = 1). The infection was determined and quantified by Operetta High Content Imaging System (PerkinElmer). Error bars represent the SD of the mean from one representative experiment with three biological replicate samples and this experiment was repeated three times. ns, no significance; *, 0.01 < *P* < 0.05; ***P < 0.001. Significance assessed by one-way ANOVA.

Although the overall protein sequences of ACE2 were largely conserved across all tested species (71 to 100% identity compared with human ACE2) (*SI Appendix*, Fig. S9), our data showed that high sequence identity does not necessarily correlate with its function to support virus entry. For example, as shown in Figs. 3 and 4, ACE2 orthologs from the New World monkeys marmoset (#11), tufted capuchin (#12), and squirrel monkey (#13) had limited or undetectable ability to mediate SARS-CoV-2 entry, despite sharing 92 to 93% identity with human ACE2. In contrast, the ACE2 proteins from *Bos taurus* (cattle, #26) or *Sus scrofa* (pig, #18) efficiently facilitated virus entry, even with 78% or 81% identity, respectively, to human ACE2 (*SI Appendix*, Fig. S9). Thus, we hypothesized that changes in critical residues in ACE2 proteins from New World monkeys may restrict viral entry.

New World monkeys are widely used in biomedical research. Our results showed that their ACE2 proteins do not bind SARS-CoV-2 S protein and do not promote virus entry, which is in line with a recent finding that marmosets are resistant to SARS-CoV-2 infection (12). To identify the genetic determinants within ACE2 orthologs from New World monkeys that restrict viral entry, we first analyzed the ACE2 protein residues that contact the S protein, especially those that form hydrogen bonds or salt bridges with the S protein, such as Q24, D30, E35, E37, D38, Y41, Q42, Y83, K353, and R393 (22, 26, 27). When comparing these with orthologs that support SARS-CoV-2 entry, we found that residues at the ACE2–S interface in New World monkeys only differed at H41 and E42 (Figs. 1*C* and 4*A*). The hydroxyl group of the Tyr (Y) at human ACE2 position 41 forms hydrogen bonds with the side-chain oxygen atom of T500 and side-chain nitrogen atom of N501 in the SARS-CoV-2 S protein. The side-chain nitrogen atom of Q42 of human ACE2 forms hydrogen bonds with the main-chain oxygen atom of G446 and side-chain hydroxyl group of Y449 of the SARS-CoV-2 S protein. Changes at these two consecutive residues, 41 and 42, may disrupt critical hydrogen-bonding interactions and thus impair the binding of New World monkey ACE2 with the SARS-CoV-2 S protein (Fig. 4 *A*, *Right*).

To directly uncover the molecular basis for the inability of New World monkey ACE2 to function as a SARS-CoV-2 receptor, we humanized marmoset and tufted capuchin ACE2 by mutating the ACE2 orthologs at positions 41 and 42 into Y and Q, respectively (Fig. 4*A*). These WT or humanized ACE2 orthologs were then transduced into A549 cells, and immunoblotting assay or cell surface immunofluorescent assay confirmed all the ACE2 proteins had comparable levels of expression and cell surface localization (Fig. 4*B* and *SI Appendix*, Fig. S10*A*). Subsequently, those cells were incubated with S1-Fc protein to determine its binding with ACE2 variants or infected with SARS-CoV-2 (MOI = 1) to assess the capabilities of the humanized orthologs to facilitate viral entry in the context of infection (Fig. 2*A*). Consistent with our previous results (Fig. 2*B* and *SI Appendix*, Fig. S5), negligible binding of ACE2 orthologs of marmoset (#11) or tufted capuchin (#12) with S1-Fc could be detected. However, single-mutation H41Y or E42Q could dramatically increase the binding of marmoset (25.9% or 52.9%) or tufted capuchin (56.9% or 61.8%) ACE2 orthologs with S1-Fc; double-mutations YQ (H41Y and E42Q) further strengthen their binding with S1-Fc (87.3% or 87.6%, respectively) (Fig. 4*C*); in parallel to the authentic virus infection, at 48 h postinfection the complemented A549 cells were subjected to immunofluorescent staining for ACE2 ortholog protein and viral nucleocapsid for quantification of the infection efficiency by the Operetta High Content Imaging System. As we observed before and consistent with our binding data, A549 cells expressing ACE2 orthologs from marmoset (#11) or tufted capuchin (#12) were nonpermissive to SARS-CoV-2 infection (Fig. 4 *D* and *E*). Single-mutation H41Y or E42Q could dramatically render the receptor activity of marmoset or tufted capuchin ACE2 orthologs, and double-mutations YQ (H41Y and E42Q) further increased the A549 cells permissiveness to infection (Fig. 4 *D* and *E*), demonstrating that altering the residues at position 41 or 42 into human counterparts confers the ability of New World monkeys ACE2 orthologs of binding to SARS-CoV-2 spike protein and mediating viral entry. A similar phenomenon was observed in HeLa cells (*SI Appendix*, Fig. S10*B*), which could exclude the cell-specific effect in A549 cells. Thus, our analysis identifies the genetic determinants of ACE2 in New World monkeys necessary for the protein’s function as the SARS-CoV-2 cellular receptor and provides insight into the species-specific restriction of viral entry.

## Discussion

To prevent the zoonotic transmission of SARS-CoV-2 to humans, the identification of animal reservoirs or intermediate hosts of SARS-CoV-2 is of great importance. Recently, a coronavirus was identified in pangolins with 90% sequence identity compared to SARS-CoV-2 (33, 34). However, the result of such phylogenetic analysis does not necessarily support the notion that pangolins are indeed an intermediate host of SARS-CoV-2. The host range and animal reservoirs of SARS-CoV-2 remain to be explored.

For the cross-species transmission of SARS-CoV-2 from intermediate hosts to humans, the virus needs to overcome at least two main host genetic barriers: the specificity of the viral S protein–ACE2 receptor interactions and the ability to escape the host’s antiviral immune response. The interaction of a virus with its host cell receptor is the first step to initiate virus infection and is a critical determinant of host species range and tissue tropism. SARS-CoV-2 uses the cellular receptor ACE2 in a species-specific manner: Human or dog ACE2 can support virus entry, whereas mouse ACE2 cannot (5). To explore possible SARS-CoV-2 animal reservoirs and intermediate hosts, we analyzed ACE2 genes from 295 vertebrates, particularly mammals. Our results suggest that ACE2 orthologs are largely conserved across vertebrate species, indicating the importance of its physiological function. Notably, we also found that ACE2 orthologs from a wide range of mammals could act as a functional receptor to mediate SARS-CoV-2 infection when ectopically expressed in A549 cells, suggesting that SARS-CoV-2 may have a diverse range of hosts and intermediate hosts, consistent with a recent study that conducted a deep phylogenetic analysis of the ACE2 orthologs propensity to bind with the SARS-CoV-2 S protein and predicted a broad host range of SARS-CoV-2 (21).

It is of note that our findings are based on a functional study of ACE2 proteins by quantitative analysis of S–ACE2 interaction and authentic SARS-CoV-2 infection. Our results are consistent with recent in vivo findings that ferrets, cats, dogs, and Old World monkeys are susceptible to SARS-CoV-2 infection, but not marmosets, which are New World monkeys (12–14). Compared with previous studies (25, 35), which included 12 to 14 ACE2 orthologs, our study tested 48 ACE2 orthologs, covering 9 orders—including Primates, Lagomorpha, Rodentia, Pholidota, Carnivora, Diprotodontia, Perissodactyla, Cetartiodactyla, and Chiroptera—and our results represent the reference to identify the potential intermediates and viral reservoir.

Interestingly, our study demonstrates that ACE2 orthologs from three New World monkey species—marmoset (#11), tufted capuchin (#12), and squirrel monkey (#13)—do not support SARS-CoV-2 entry, even though they share higher sequence identity with human ACE2 than that of rabbit (#14), dog (#35), or cattle (#26), indicating that sequence identity with human ACE2 does not necessarily correlate with its function to support virus entry. We identified specific residues (H41 and E42) within ACE2 that restrict SARS-CoV-2 in these species. Substituting single or both critical amino acids with those found in human ACE2 rendered these ACE2 orthologs able to support SARS-CoV-2 entry. However, koala or mouse ACE2—at positions of 41 and 42 are Y and Q, which is the same with that of human ACE2 (Fig. 1*C*)—still cannot mediate SARS-CoV-2 entry. These observations suggests that species-specific restriction mechanisms exist regulating ACE2 ortholog receptor activity, and also demonstrate that Y41 and Q42 of ACE2 are necessary, but not sufficient for its receptor activities, which is further supported by a deep mutagenesis study of ACE2 (36). It is also interesting that ferret or mink ACE2 bind SARS-CoV-2 with limited affinity (Fig. 2*B* and *SI Appendix*, Fig. S3), but they could mediate authentic virus entry of the cells, albeit less efficiently than human ACE2 (Fig. 3). Recently, mink-associated SARS-CoV-2 variants were identified in Denmark, which circulate in mink farms and could transmit to humans (37). Of note are the “Cluster 5” variants, which were isolated from patients: del69–70 (a deletion of the histidine and valine residues at the 69th and 70th position), Y453F, I692V, and M1229I, in which Y453F is localized in the spike RBD (37). It is conceivable that after SARS-CoV-2 transmission to mink, and the adaptive mutations that could dramatically enhance the interaction of spike with mink ACE2 were selected to adapt the new host, the mink associated virus variants could circulate among the mink population and then spill over to humans.

It has to be mentioned that our study had several limitations. First, the host range or specificity of a virus is often limited for several reasons: for example, the lack of host factors the virus depends on or the incompatibility of these factors’ orthologs in different species. Alternatively, but not necessarily mutually exclusive, the ability to evade the antiviral immune response of a given host can also shape the species tropism of viruses (8, 38). Second, our study is based on the assessment of viral receptor ACE2 functionalities as the evidence to reveal the potential host range, whereas the serine protease TMPRSS2, priming the viral spike for entry via a direct plasma membrane route, could be another potential host factor to determine SARS-CoV-2 host range. However, TMPRSS2 proteases from different species may work with comparable efficiencies, as the WT mice are insusceptible to SARS-CoV-2 infection, while the human ACE2 transgenic mouse can be infected (10, 39–41), which suggests that mouse TMPRSS2 is sufficiently efficient in priming viral spike. Third, we retrieved ACE2 ortholog sequences from the NCBI databases for chemical synthesis to generate the expression constructs, and we did not have experimental evidence that the ACE2 sequences in each species are a functional gene to encode the protein. Given the important physiological functions of ACE2, it is conceivable that the ACE2 orthologs could express and encode the functional proteins at least in the species in this study. Fourth, we predicted viral receptor activity of ACE2 orthologs by analyzing the conservation of five amino acids in K31 and K353 hotspots (*Materials and Methods*), and the cutoff might be too stringent, which could exclude potential ACE2 orthologs with receptor activities. Fifth, as evidenced by the New World monkey and koala ACE2s (Figs. 1*C* and 4*A*), the conservation residues in K31 and K353 hotspots could also not completely distinguish ACE2’s viral receptor function, which suggested that other critical genetic determinants exist. Y41 and Q42 have been demonstrated as the genetic restriction of New World monkey ACE2 viral receptor activities (Fig. 4 *D* and *E*); however, the genetic basis of koala ACE2 unable to function as a receptor was not known. Due to those limitations, we urge caution not to overinterpret the results of this study.

In summary, we systematically assessed the functionality of ACE2 orthologs from 48 mammalian hosts using the authentic SARS-CoV-2 virus, which provides insight into the potential host range and cross-species transmission of this virus. It also suggests that SARS-CoV-2 might be much more widely distributed than previously thought, underscoring the necessity of monitoring susceptible hosts, especially their potential for causing zoonosis to prevent future outbreaks.

## Materials and Methods

### Prediction of ACE2 Orthologs’ Receptor Activities.

Based on the structures of SARS-CoV-2 S protein complexed with human ACE2, five critical amino acid residues of human ACE2 (31K, 35E, 38D, 82M, or 353K) constitute two virus-binding hotspots (31K hotspot and 353K hotspot) that are indispensable for interaction with S protein and viral entry (22). The 31K hotspot consists of a salt bridge between 31K and 35E, and the 353K hotspot consists of a salt bridge between 353K and 38D; 82M is critical to stabilize 31K hotspot through hydrophobic interaction with 486F of SARS-CoV-2 RBD (22, 28). According to Li and colleagues analysis of the conservation of these five critical residues in known susceptible species (31K/T, 35E/K, 38D/E, 82T/M/N, and 353K) (23), we applied this predictive framework to predict the potential ACE2 orthologs possessing viral receptor activity. The ACE2 orthologs completely applied to the 31K/T, 35E/K, 38D/E, 82T/M/N, and 353K rule were predicted as potential functional receptors.

### Phylogenetic Analysis and Sequence Alignment.

The amino acid sequences of 80 ACE2 orthologs with potential viral receptor activity were exported from the NCBI nucleotide database and were then passed into MEGA-X (v10.05) software for further analysis. The alignment was conducted using the MUSCLE algorithm (42). Then the alignment file was used to construct the phylogenetic tree (neighbor-joining option of the MEGA-X with default parameter).

### Surface ACE2 Binding with S1-Fc Assay.

A549 cells were transduced with bicistronic lentiviruses (pLVX-IRES-zsGreen) expressing the ACE2 from different species and the zsGreen reporter for 48 h. The cells were collected with TrypLE (Thermo #12605010) and washed twice with cold PBS. Live cells were incubated with the recombinant protein, S1 domain of SARS-CoV-2 spike C-terminally fused with Fc (Sino Biological #40591-V02H; 1 μg/mL) at 4 °C for 30 min. After washing, cells were stained with goat anti-human IgG (H + L) conjugated with Alexa Fluor 647 (Thermo #A21445; 2 μg/mL) for 30 min at 4 °C. Cells were then washed twice and subjected to flow cytometry analysis (Thermo, Attune NxT). Binding efficiencies are expressed as the percent of cells positive for S1-Fc among the zsGreen positive cells (ACE2-expressing cells).

### Analysis of SARS-CoV-2 Infection by High-Content Imaging System.

A549 cells were transduced with lentiviruses expressing the ACE2 from different species for 48 h. Cells were then infected with nCoV-SH01(SARS-CoV-2) at an MOI of 1 for 1 h, washed three times with PBS, and incubated in 2% FBS culture medium for 48 h for viral antigen staining. Cells were fixed with 4% paraformaldehyde in PBS, permeablized with 0.2% Triton X-100, and incubated with the rabbit polyclonal antibody against SARS-CoV nucleocapsid protein (Rockland, 200-401-A50; 1 μg/mL) and mouse anti-FLAG M2 antibody (Sigma-Aldrich #1804; 1 μg/mL) at 4 °C overnight. After three washes, cells were incubated with the secondary goat anti-rabbit antibody conjugated with Alexa Fluor 555 (Thermo #A32732; 2 μg/mL) and goat anti-mouse antibody conjugated with Alexa Fluor 647 (Thermo #A21235; 2 μg/mL) for 2 h at room temperature, followed by staining with DAPI. Images were collected using an Operetta High Content Imaging System (PerkinElmer). For high-content imaging, three biological replicates for each species on different plates were scanned and five representative fields were selected for each well of 96-well plates. Image analysis was performed using the PerkinElmer Harmony high-content analysis software 4.9. ACE2-transduced cells were automatically identified by DAPI (nuclei) and zsGreen (cytoplasm, the lentiviral vector containing the ACE2 orthologs also expressed zsGreen). Mean fluorescent intensity (MFI) of the Alexa 647 (ACE2 orthologs) and Alexa 555 (viral nucleocapsid) channels were subsequently calculated for each cell. Cells with an Alexa 647 channel MFI > 1,000 were gated as ACE2^+^ cells. ACE2^+^ cells with an Alexa 555 channel MFI >1,600 were gated as SARS-CoV-2–infected cells. Formula SARS-CoV-2–infected cells/ACE2^+^ cells was outputted as infection efficiency.

### Statistics Analysis.

One-way ANOVA with Tukey’s honestly significant difference test was used to test for statistical significance of the differences between the different group parameters. *P* values of less than 0.05 were considered statistically significant.

## Supplementary Material

Supplementary File

Supplementary File

Supplementary File

## Data Availability

All study data are included in the article and supporting information.
