# Supplementary material for: Functional and genetic analysis of viral receptor ACE2 orthologs reveals a broad potential host range of SARS-CoV-2
Source: Proc Natl Acad Sci U S A. 2021 Mar 3;118(12):e2025373118. doi: 10.1073/pnas.2025373118 (PMC8000431; doi:10.1073/pnas.2025373118)
Supplement: Supplementary File [file pnas.2025373118.sapp.pdf]

PNAS
www.pnas.org

# Supplementary Information for

## Functional and Genetic Analysis of Viral Receptor ACE2 Orthologs Reveals a Broad Potential Host Range of SARS-CoV-2

Yinghui Liu[1*], Gaowei Hu[2 *], Yuyan Wang[2 *], Wenlin Ren[1*], Xiaomin Zhao[1*], Fansen Ji[1], Yunkai Zhu[2], Fei Feng[2], Mingli Gong[1], Xiaohui Ju[1], Yuanfei Zhu[2], Xia Cai[2], Jun Lan[3], Jianying Guo[1], Min Xie[1], Lin Dong[1], Zihui Zhu[1], Jie Na[1], Jianping Wu[4,5], Xun Lan[1], Youhua Xie[2], Xinquan Wang[3,6], Zhenghong Yuan[2 †], Rong Zhang[2 †], Qiang Ding[1,6 †]

[†]To whom correspondence should be addressed: Qiang Ding: qding@tsinghua.edu.cn; Rong Zhang: rong_zhang@fudan.edu.cn; and Zhenghong Yuan: zhyuan@shmu.edu.cn.

**This PDF file includes:**

SI Materials and Methods
Figures S1 to S10
SI References

**Other supplementary materials for this manuscript include the following:**

Datasets S1 to S2

## SI Materials and Methods

**Cell cultures and SARS-CoV-2 virus.** HEK293T cells (American Tissue Culture Collection, ATCC, Manassas, VA, CRL-3216), HeLa cells (a gift from Dr. Nian Liu, Tsinghua University), Vero E6 (Cell Bank of the Chinese Academy of Sciences, Shanghai, China) and A549 (ATCC) were maintained in Dulbecco's modified Eagle medium (DMEM) (Gibco, NY, USA) supplemented with 10% (vol/vol) fetal bovine serum (FBS), 10mM HEPES, 1mM sodium pyruvate, 1×non-essential amino acids, and 50 IU/ml penicillin/streptomycin in a humidified 5% (vol/vol) $CO_2$ incubator at 37°C. Cells were tested routinely and found to be free of mycoplasma contamination. The SARS-CoV-2 strain nCoV-SH01 (GenBank accession no. MT121215) was isolated from a COVID-19 patient and propagated in Vero E6 cells for use. All experiments involving virus infections were performed in the biosafety level 3 facility of Fudan University following the regulations.

**Plasmids.** The cDNAs encoding ACE2 orthologs (Dataset S1) were codon-optimized and synthesized by GenScript and cloned into pLVX-IRES-zsGreen1 vectors (Catalog No. 632187, Clontech Laboratories, Inc) with a C-terminal FLAG tag. Human cDNA encoding TMPRSS2 were synthesized by GenScript and cloned into pLVX-IRES-Puro vectors (Catalog No. 632183, Clontech Laboratories, Inc). ACE2 mutants were generated by Quikchange (Stratagene) site-directed mutagenesis. All of the constructs were verified by Sanger sequencing.

**Lentivirus production.** Vesicular stomatitis virus G protein (VSV-G) pseudotyped lentiviruses expressing ACE2 orthologs tagged with FLAG at the C-terminus were produced by transient co-transfection of the third-generation packaging plasmids pMD2G (Addgene #12259) and psPAX2 (Addgene #12260) and the transfer vector with VigoFect DNA transfection reagent (Vigorous) into HEK293T cells. The medium was changed 12 h post transfection. Supernatants were collected at 24 and 48h after transfection, pooled, passed through a 0.45-μm filter, and frozen at -80°C.

**Western blotting.** Sodium dodecyl sulfate-polyacrylamide gel electrophoresis (SDS-PAGE) immunoblotting was performed as follows: After trypsinization and cell pelleting at 2,000 × g for 10 min, whole-cell lysates were harvested in RIPA lysis buffer (50 mM Tris-HCl [pH 8.0], 150mM NaCl, 1% NP-40, 0.5% sodium deoxycholate, and 0.1% SDS) supplemented with protease inhibitor cocktail (Sigma). Lysates were electrophoresed in 12% polyacrylamide gels and transferred onto nitrocellulose membrane. The blots were blocked at room temperature for 0.5 h using 5% nonfat milk in 1×phosphate-buffered saline (PBS) containing 0.1% (v/v) Tween 20. The blots were exposed to primary antibodies anti-β-Tubulin (CW0098, CWBIO), anti-TMPRSS2 (ab109131, Abcam) or anti-FLAG (F7425, Sigma) in 5% nonfat milk in 1× PBS containing 0.1% Tween 20 for 2 h. The blots were then washed in 1× PBS containing 0.1% Tween 20. After 1h exposure to HRP-conjugated secondary antibodies, subsequent washes were performed and membranes were visualized using the Luminescent image analyzer (GE).

**Cell surface staining of ACE2 orthologs.** The procedure of this experiment was as described previously (1). Briefly, A549 cells transduced with ACE2 orthologs were collected and washed with PBS twice. Cells were subsequently incubated with rabbit polyclonal antibody (Sino Biological Inc. China, Cat: 10108-T24) against ACE2 orthologs at 4°C for 30 min. After twice washing with PBS, cells were stained with 2μg/mL goat anti-rabbit IgG (H+L) conjugated with Alexa Fluor 568 (Thermo Fisher Scientific #A11036) for 30 min at 4°C. After two additional washes, cells were fixed with 2 % PFA for 10 min at room temperature. Cells were then washed twice and subjected to flow cytometry analysis. The percentage of cell surface localization is defined as the percent of cells positive for cell surface ACE2 among the zsGreen positive cells (ACE2 expressing cells).

**Surface Plasmon Resonance (SPR) experiments.** The SARS-CoV-2 RBD (residues Try333–Pro527) and the N-terminal peptidase domain of human, ferret, mink or mouse ACE2 (residues Ser19–Asp615) were expressed using the Bac-to-Bac baculovirus system (Invitrogen) as described previously (2). The recombinant ACE2 proteins were immobilized to a CM5 sensorchip (GE Healthcare) to a level of ~540 response units (RUs) using a Biacore T200 (GE Healthcare) instrument and a running buffer (10 mM HEPES pH 7.2, 150 mM NaCl and 0.05% Tween 20). SARS-CoV-2-RBD were flowed through with a concentration ranging from 800 to 50 nM. The resulting data were fit to a 1:1 binding model using Biacore Evaluation Software (GE Healthcare).

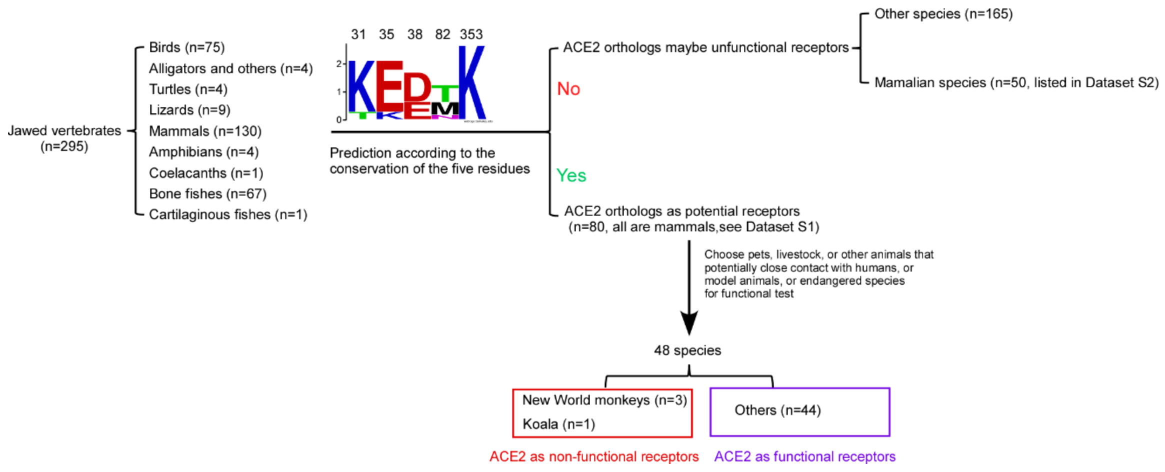


**Supplemental Figure 1. A pipeline for selection of ACE2 orthologs for functional analysis.** 295 ACE2 orthologs are downloaded from NCBI. According to the structure of human ACE2 and SARS-CoV-2 spike protein, five critical amino acid residues of human ACE2 (31K, 35E, 38D, 82M or 353K) constitute two virus-binding hotspots (K31 hotspot and K353 hotspot) that are indispensable for interaction with S protein and viral entry. Based on this structural information and conservation of these five critical residues in known susceptible species (31K/T, 35E/K, 38D/E, 82T/M/N and 353K) as reported. 80 ACE2 orthologs contained the relevant residues at those positions among 295 orthologs were predicted to function as SARS-CoV-2 receptors. Among the 80 orthologs, 48 ACE2 orthologs were chose from species which are in close contact with humans, model animals in biomedical research or endangered species, for further analysis to determine their receptor activities. Among the 48 orthologs tested, 44 ACE2 orthologs could function as viral receptors, meanwhile, 3 orthologs of New would monkeys and 1ortholog of koala could not support virus entry.

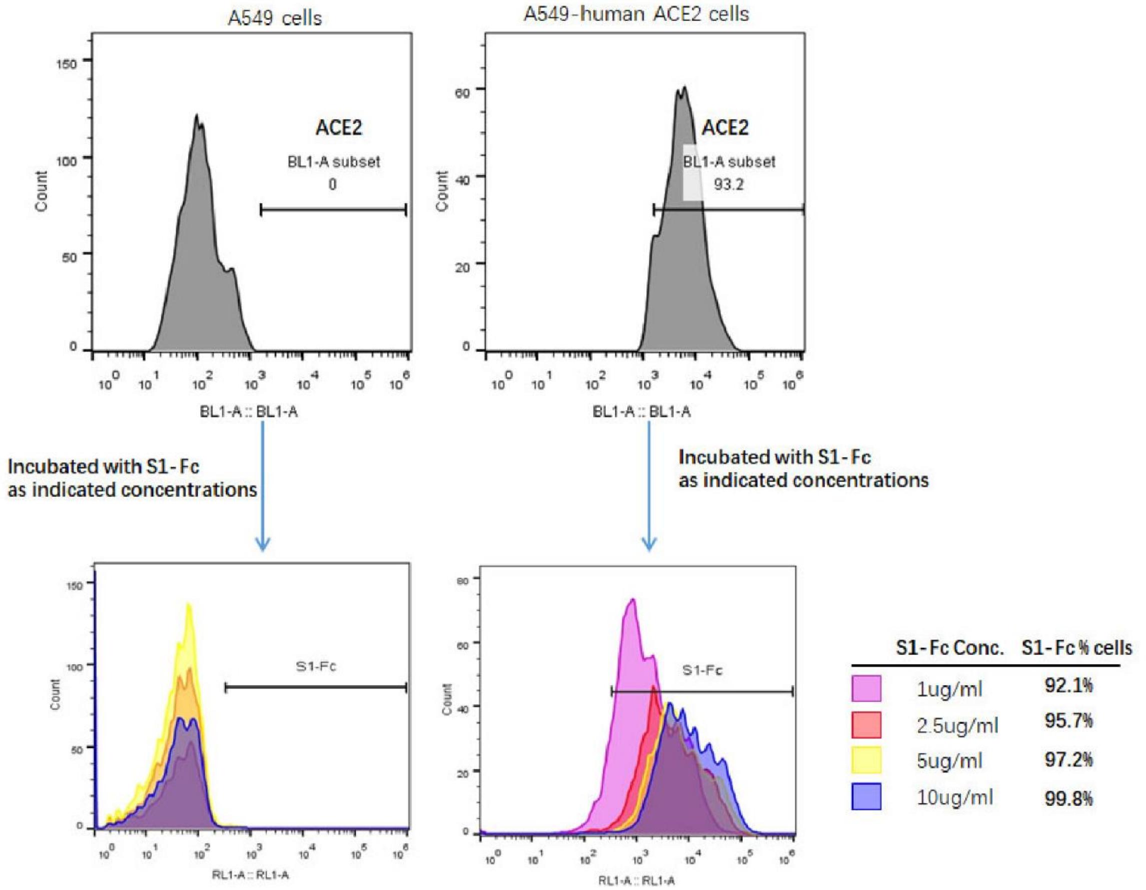


**Supplemental Figure 2. Determination of the S1-Fc doses for detecting interaction of ACE2 with SARS-CoV-2 spike.** Human ACE2 was cloned into pLVX-IRES-zsGreen vector for production of lentivirues to transduce the A549 cells and the transduction efficiency (93.2%) was quantified by flow cytometry. The A549 cells or A549-human ACE2 cells were incubated with different concentration of recombinant S1-Fc protein as indicated, and then stained with goat anti-human IgG (H + L) conjugated to Alexa Fluor 647 for flow cytometry analysis. Binding efficiencies are expressed as the percent of cells positive for S1-Fc among the ACE2 expressing cells (zsGreen positive) from one representative experiment with three replicates.

# Supplemental Figure 3

A

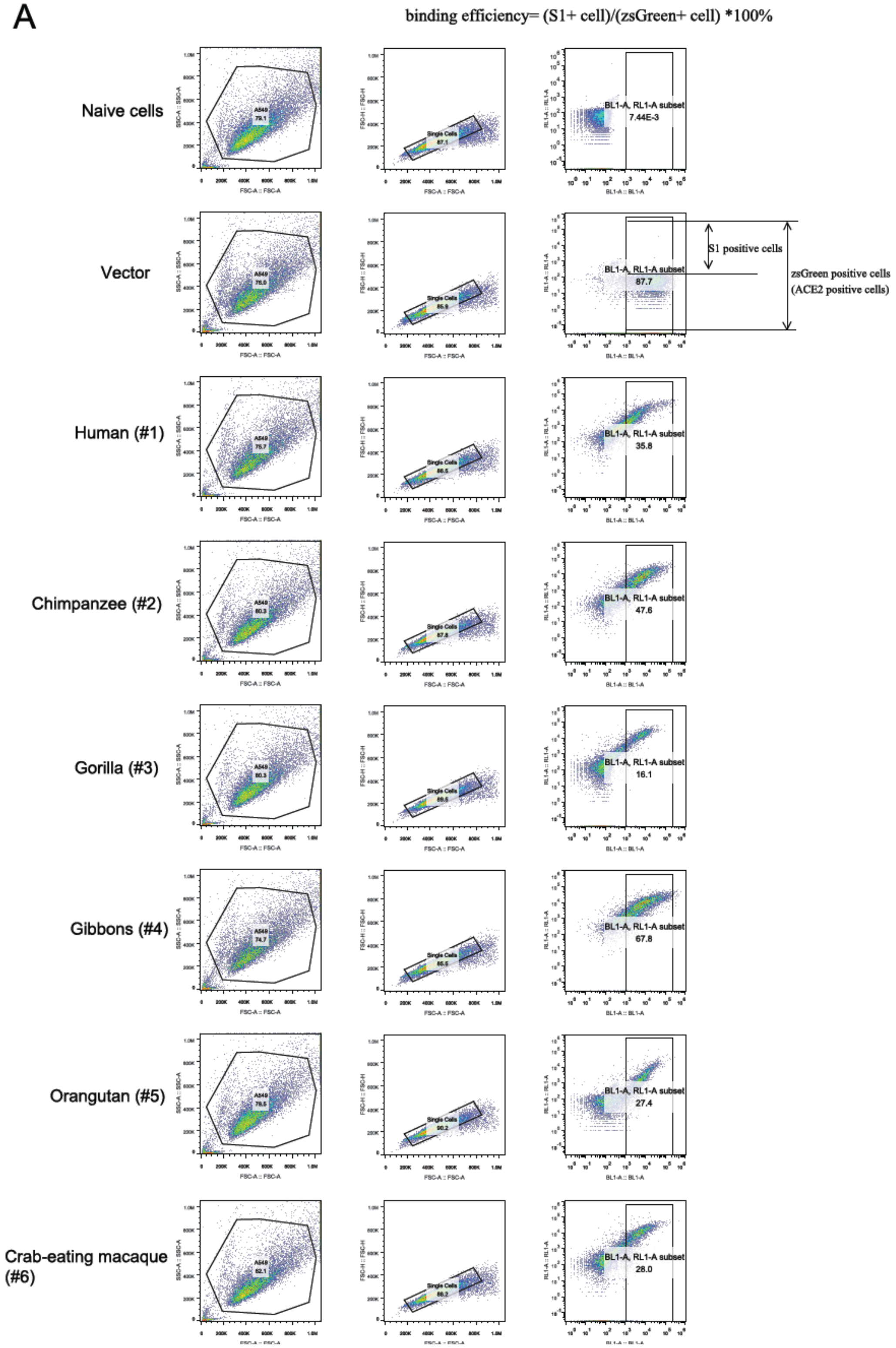

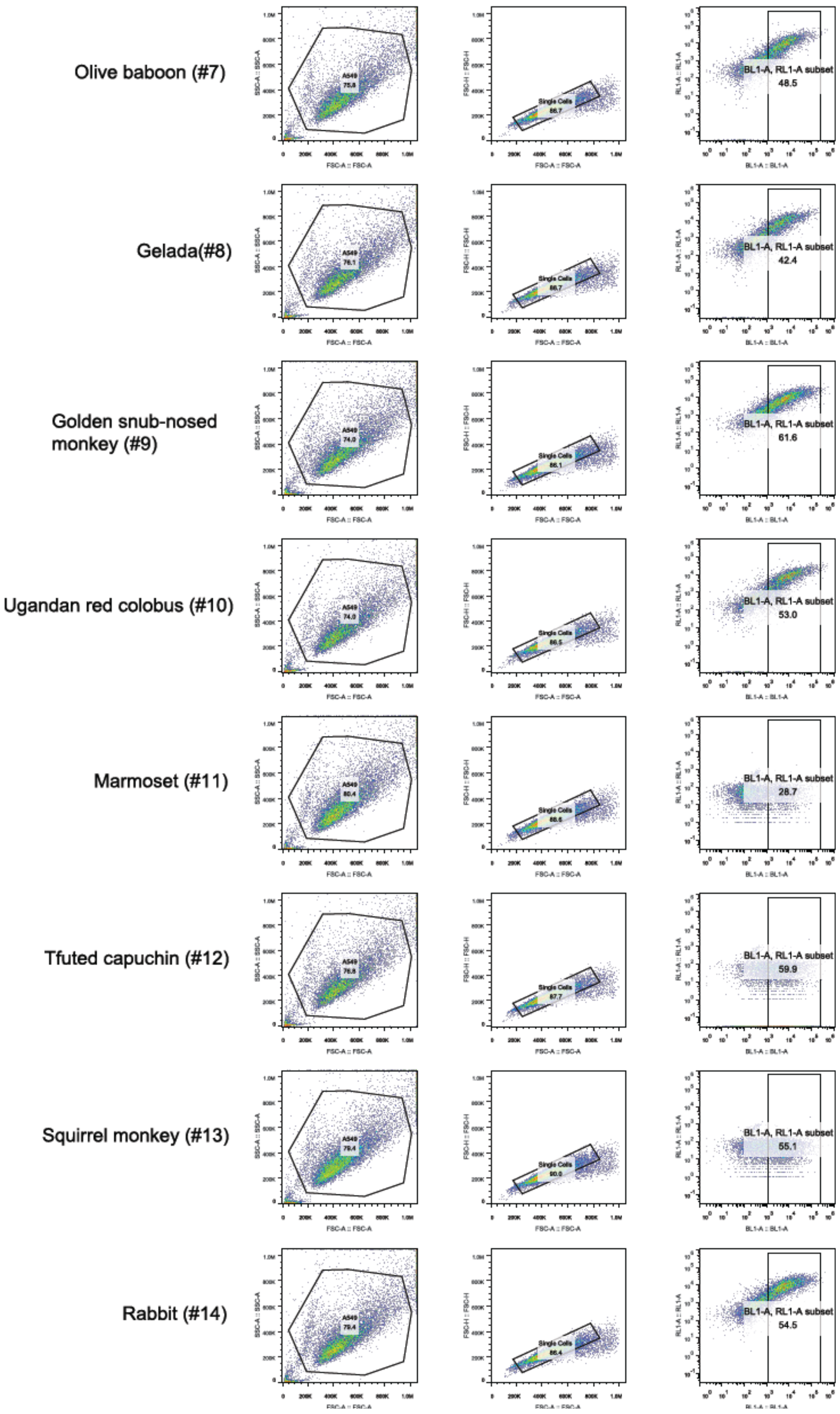

Olive baboon (#7)
BL1-A, RL1-A subset
48.5
Gelada(#8)
BL1-A, RL1-A subset
42.4
Golden snub-nosed monkey (#9)
BL1-A, RL1-A subset
61.6
Ugandan red colobus (#10)
BL1-A, RL1-A subset
53.0
Marmoset (#11)
BL1-A, RL1-A subset
28.7
Tfuted capuchin (#12)
BL1-A, RL1-A subset
59.9
Squirrel monkey (#13)
BL1-A, RL1-A subset
55.1
Rabbit (#14)
BL1-A, RL1-A subset
54.5

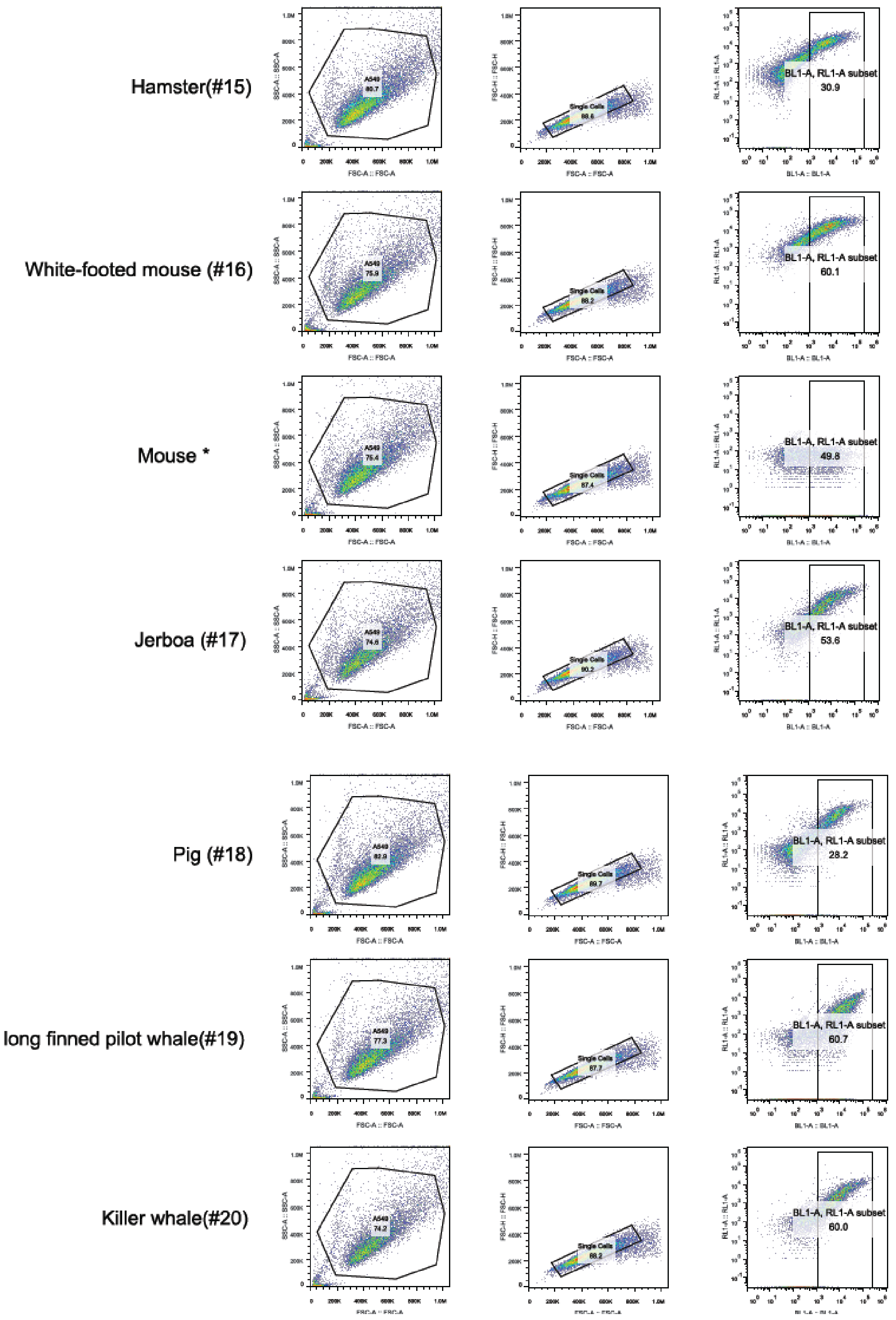
Hamster(#15)
White-footed mouse (#16)
Mouse *
Jerboa (#17)
Pig (#18)
long finned pilot whale(#19)
Killer whale(#20)

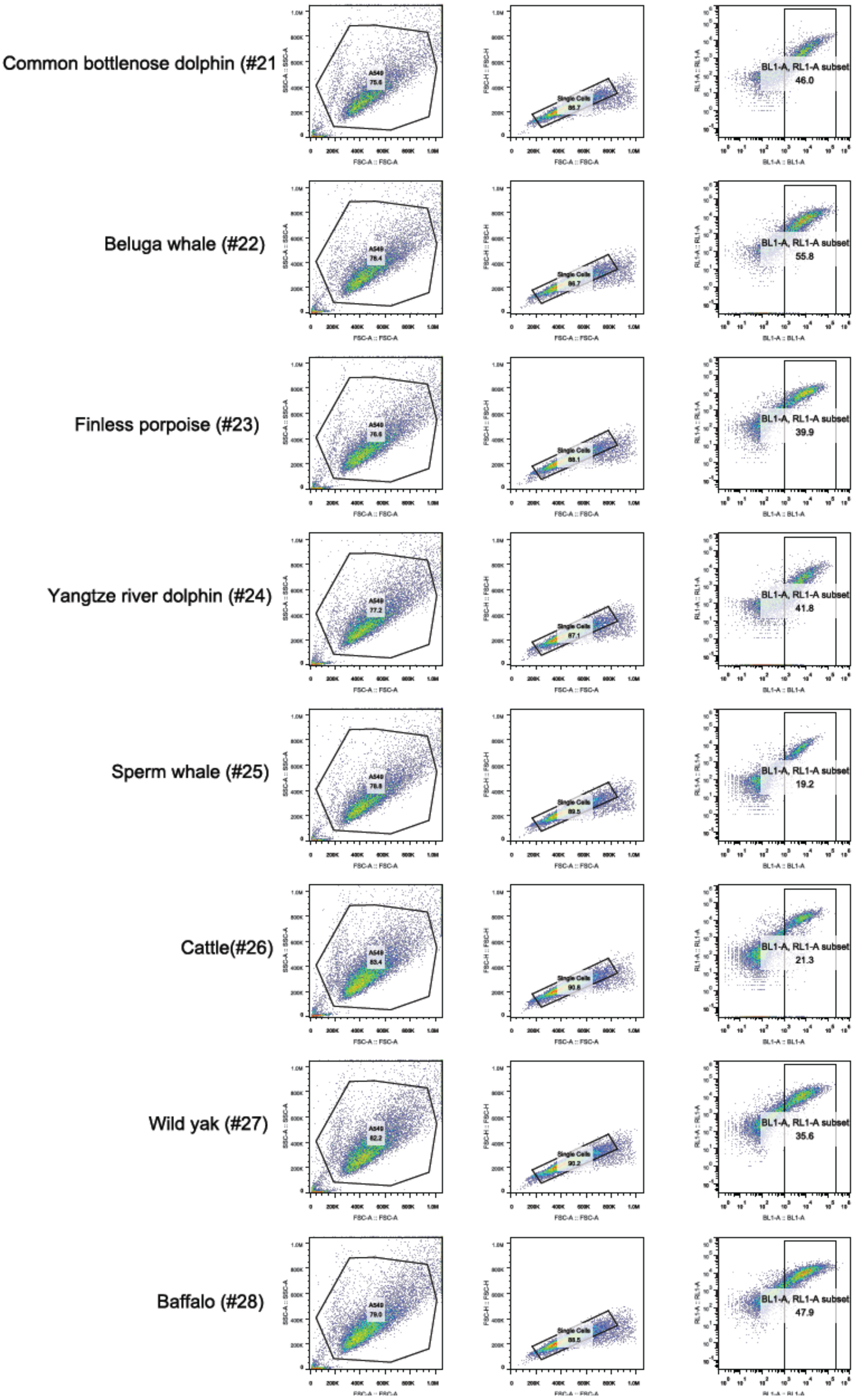
Common bottlenose dolphin (#21
BL1-A, RL1-A subset
46.0
Beluga whale (#22)
BL1-A, RL1-A subset
55.8
Finless porpoise (#23)
BL1-A, RL1-A subset
39.9
Yangtze river dolphin (#24)
BL1-A, RL1-A subset
41.8
Sperm whale (#25)
BL1-A, RL1-A subset
19.2
Cattle(#26)
BL1-A, RL1-A subset
21.3
Wild yak (#27)
BL1-A, RL1-A subset
35.6
Baffalo (#28)
BL1-A, RL1-A subset
47.9

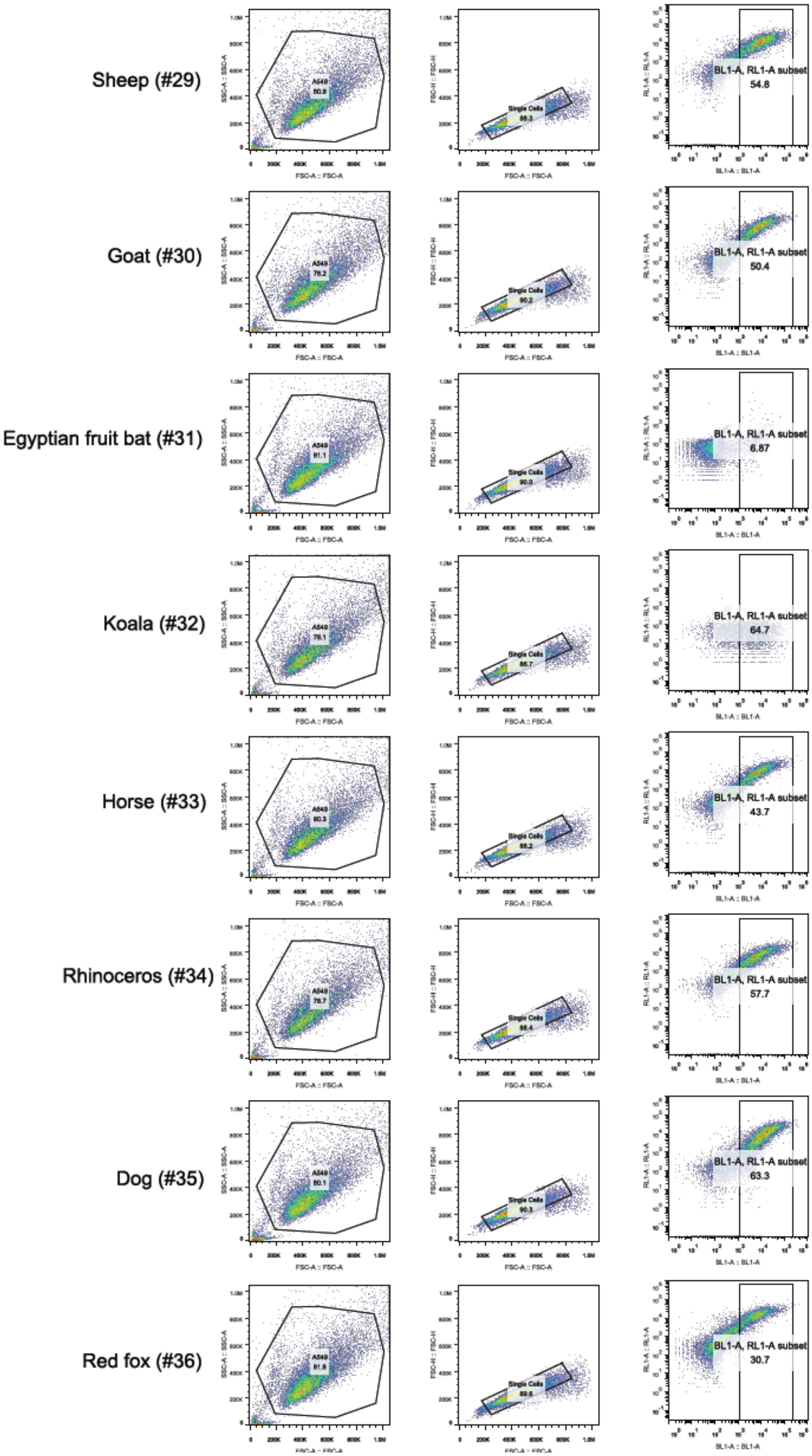

Sheep (#29)
BL1-A, RL1-A subset
54.8
Goat (#30)
BL1-A, RL1-A subset
50.4
Egyptian fruit bat (#31)
BL1-A, RL1-A subset
6.87
Koala (#32)
BL1-A, RL1-A subset
64.7
Horse (#33)
BL1-A, RL1-A subset
43.7
Rhinoceros (#34)
BL1-A, RL1-A subset
57.7
Dog (#35)
BL1-A, RL1-A subset
63.3
Red fox (#36)
BL1-A, RL1-A subset
30.7

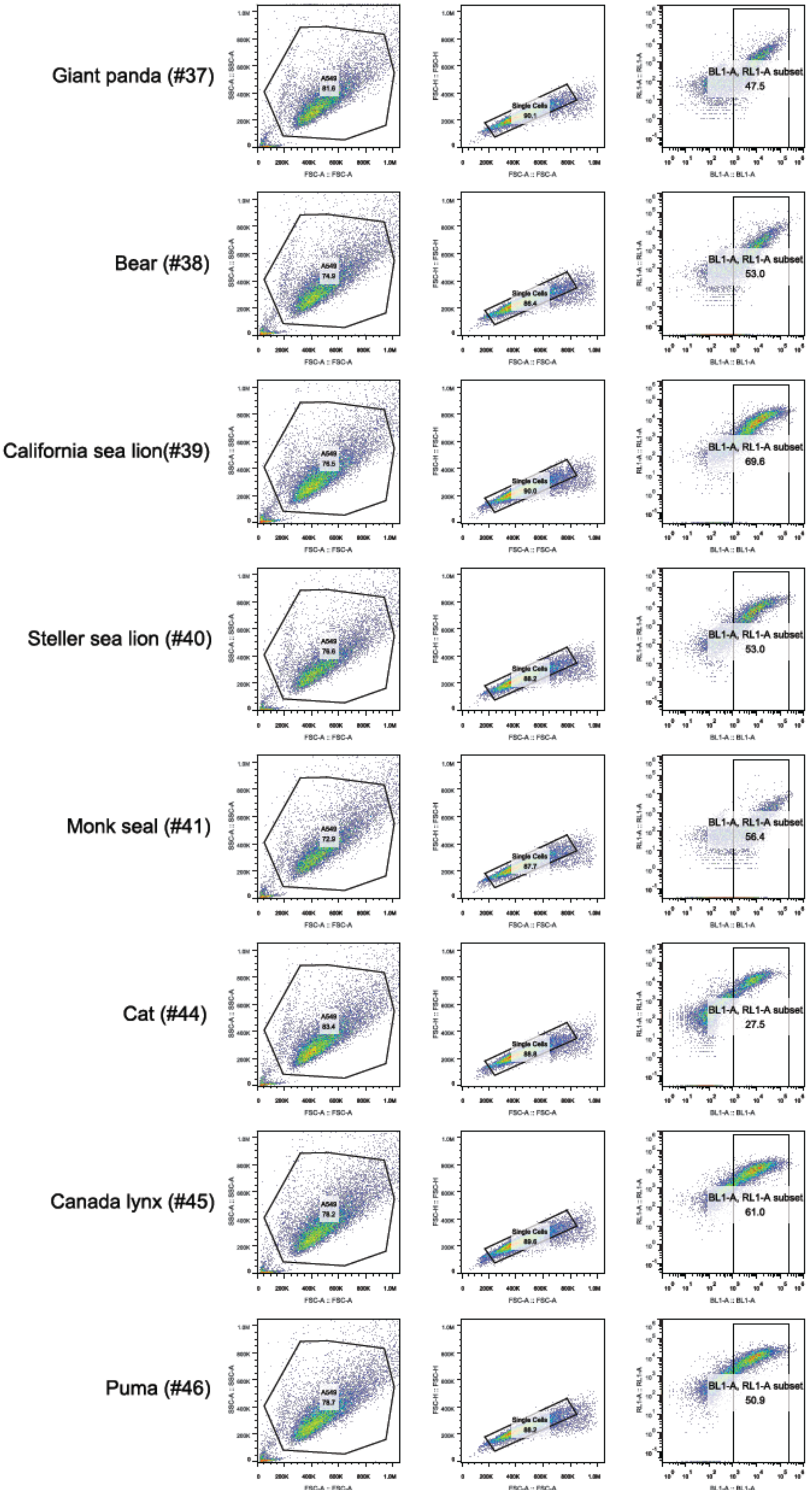
Giant panda (#37)
BL1-A, RL1-A subset
47.5
Bear (#38)
BL1-A, RL1-A subset
53.0
California sea lion(#39)
BL1-A, RL1-A subset
69.6
Steller sea lion (#40)
BL1-A, RL1-A subset
53.0
Monk seal (#41)
BL1-A, RL1-A subset
56.4
Cat (#44)
BL1-A, RL1-A subset
27.5
Canada lynx (#45)
BL1-A, RL1-A subset
61.0
Puma (#46)
BL1-A, RL1-A subset
50.9

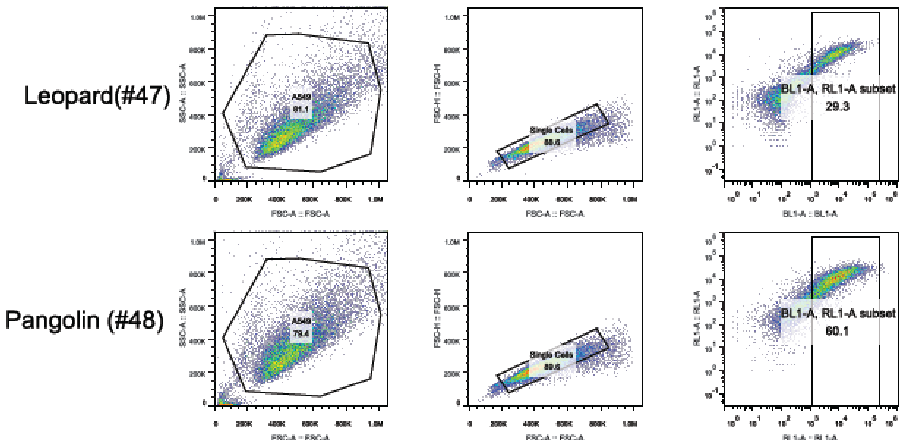

Leopard(#47)
Pangolin (#48)
A549
81.1
Single Cells
88.6
BL1-A, RL1-A subset
29.3
A549
79.4
Single Cells
89.6
BL1-A, RL1-A subset
60.1
FSC-A :: FSC-A
SSC-A :: SSC-A
FSC-H :: FSC-H
BL1-A :: BL1-A
RL1-A :: RL1-A

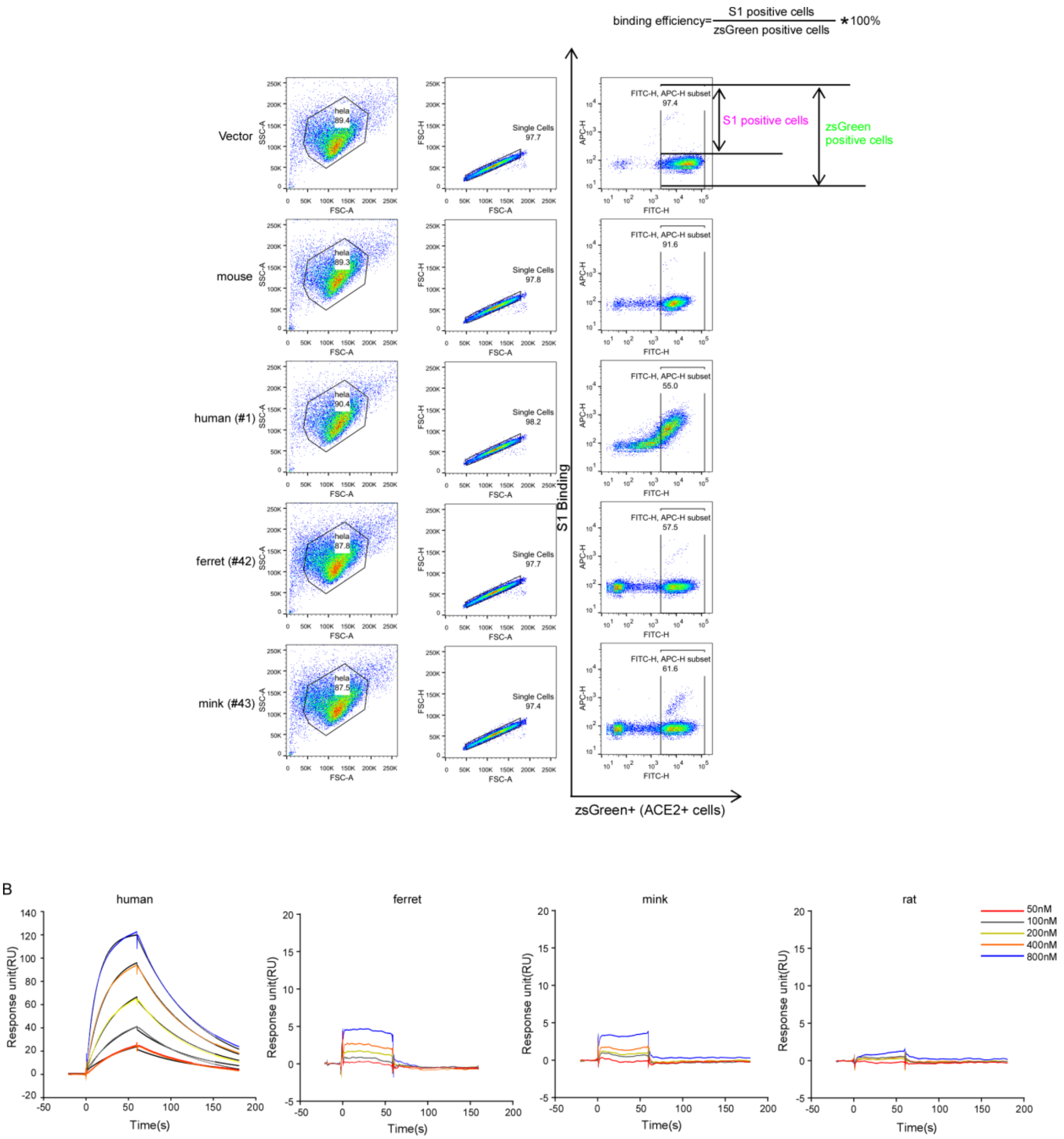


**Supplemental Figure 3. Gating strategy of cell based assay or SPR assay for determination of the binding efficiency of ACE2 orthologs with SARS-CoV-2 RBD protein.** (***A***) Main cell population was identified and gated on Forward and Side Scatter. Single cells were further gated on FSC-A and FSC-H. The gated cells were plotted by BL-1A (zsGreen, as the ACE2 expressing population) and RL-1A (S1-Fc bound population). The BL-1A positive cell population was plotted as a histogram to show the S1-Fc positive population as Fig 2*B*. The binding efficiency was defined as the percent of S1-Fc binding cells among the zsGreen positive cells. Shown are FACS plots representative of those that have been used for the calculations of binding efficiencies of

ACE2 orthologs with S1-Fc. This experiment was independently repeated three times with similar results. (***B***) The binding kinetics of ACE2 proteins (human, ferret, mink or mouse) with recombinant SARS-CoV-2 RBD were obtained using the BIAcore. ACE2 proteins were captured on the chip, and serial dilutions of RBD were then injected over the chip surface. Experiments were performed three times with similar result, and one set of representative data is displayed.

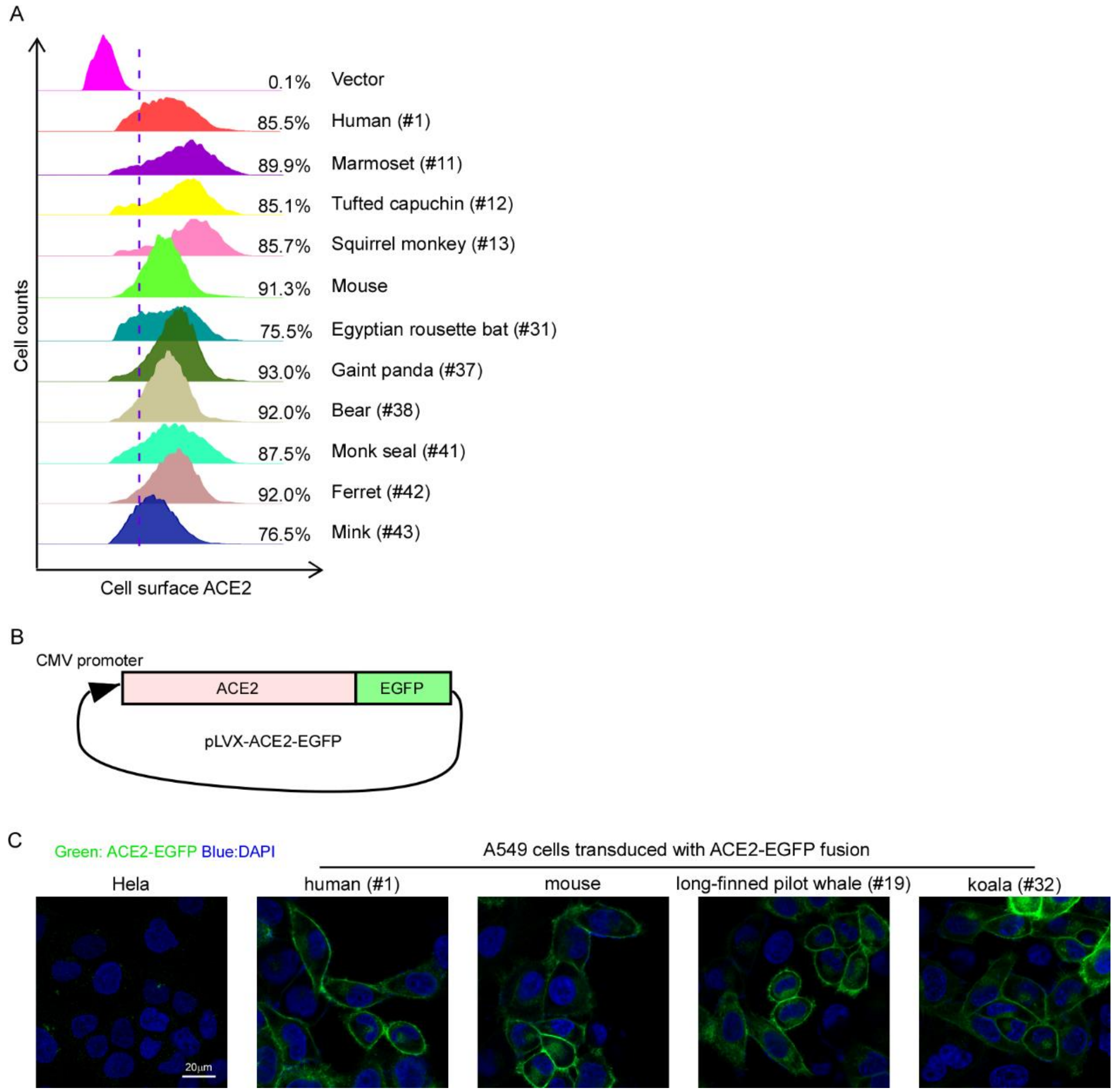


**Supplemental Figure 4**. **Cell surface localization of ACE2 orthologs.** (***A***) A549 cells transduced with lentiviruses (pLVX-IRES-zsGreen) expressing ACE2 orthologs from different species and humanized variants were incubated with rabbit polyclonal antibody (Sino Biological Inc. China, Cat: 10108-T24) against ACE2. The cells were washed and then stained with 2μg/mL goat anti-rabbit IgG (H+L) conjugated with Alexa Fluor 568 for flow cytometry analysis. The cell surface ACE2 was calculated as the percent of Alex Fluor 568 positive cells among the zsGreen positive cells. This experiment was repeated twice with similar result. (***B***) Human, mouse, long-finned pilot whale or koala ACE2 cDNA was cloned as a carboxyl terminus fusion with EGFP. (***C***) A549 cells were transduced with lentivirus expressing ACE2 ortholog-EGFP proteins in (*B*) and cells were collected, washed and counterstained with DAPI (1μg/ml). The cell images were captured with a Zeiss LSM 880 Confocal Microscope. ACE2 on cell surface was shown in the

merge images processed by ZEN3.2 software. This experiment was independently repeated twice with similar result and the representative images were shown.

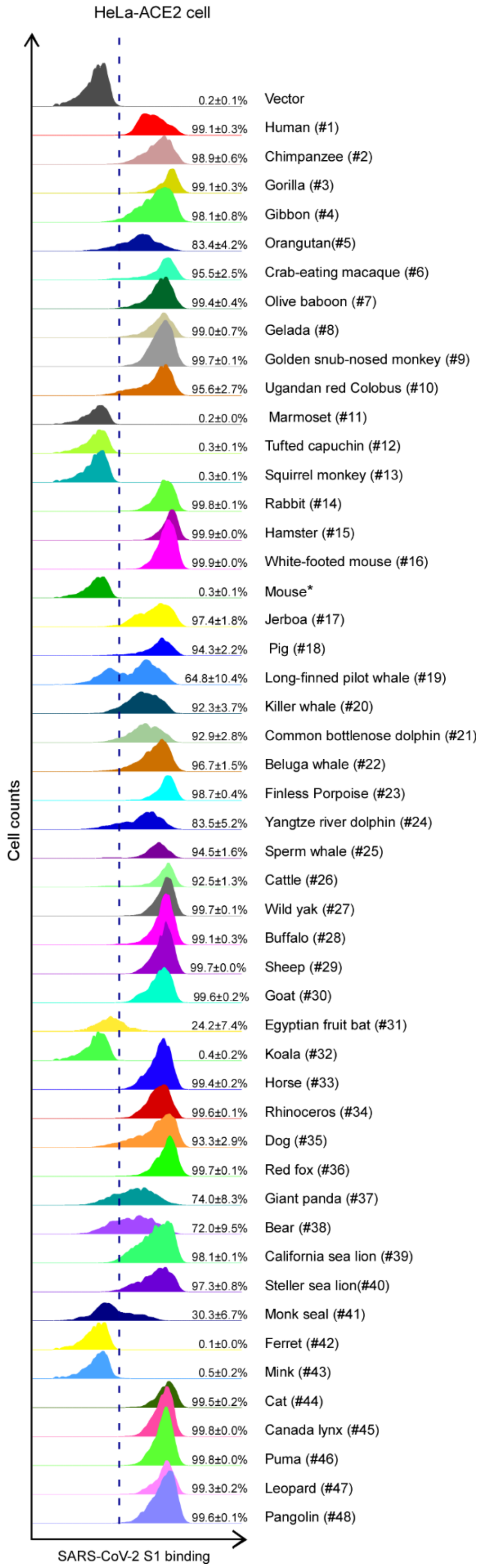
HeLa-ACE2 cell
Cell counts
SARS-CoV-2 S1 binding
0.2±0.1% Vector
99.1±0.3% Human (#1)
98.9±0.6% Chimpanzee (#2)
99.1±0.3% Gorilla (#3)
98.1±0.8% Gibbon (#4)
83.4±4.2% Orangutan(#5)
95.5±2.5% Crab-eating macaque (#6)
99.4±0.4% Olive baboon (#7)
99.0±0.7% Gelada (#8)
99.7±0.1% Golden snub-nosed monkey (#9)
95.6±2.7% Ugandan red Colobus (#10)
0.2±0.0% Marmoset (#11)
0.3±0.1% Tufted capuchin (#12)
0.3±0.1% Squirrel monkey (#13)
99.8±0.1% Rabbit (#14)
99.9±0.0% Hamster (#15)
99.9±0.0% White-footed mouse (#16)
0.3±0.1% Mouse*
97.4±1.8% Jerboa (#17)
94.3±2.2% Pig (#18)
64.8±10.4% Long-finned pilot whale (#19)
92.3±3.7% Killer whale (#20)
92.9±2.8% Common bottlenose dolphin (#21)
96.7±1.5% Beluga whale (#22)
98.7±0.4% Finless Porpoise (#23)
83.5±5.2% Yangtze river dolphin (#24)
94.5±1.6% Sperm whale (#25)
92.5±1.3% Cattle (#26)
99.7±0.1% Wild yak (#27)
99.1±0.3% Buffalo (#28)
99.7±0.0% Sheep (#29)
99.6±0.2% Goat (#30)
24.2±7.4% Egyptian fruit bat (#31)
0.4±0.2% Koala (#32)
99.4±0.2% Horse (#33)
99.6±0.1% Rhinoceros (#34)
93.3±2.9% Dog (#35)
99.7±0.1% Red fox (#36)
74.0±8.3% Giant panda (#37)
72.0±9.5% Bear (#38)
98.1±0.1% California sea lion (#39)
97.3±0.8% Steller sea lion(#40)
30.3±6.7% Monk seal (#41)
0.1±0.0% Ferret (#42)
0.5±0.2% Mink (#43)
99.5±0.2% Cat (#44)
99.8±0.0% Canada lynx (#45)
99.8±0.0% Puma (#46)
99.3±0.2% Leopard (#47)
99.6±0.1% Pangolin (#48)

**Supplemental Figure 5**. **Binding of the SARS-CoV-2 spike protein to HeLa cells transduced with ACE2 orthologs.** HeLa cells were transduced with ACE2 orthologs of the indicated species, incubated with the recombinant S1-Fc, and then stained with goat anti-human IgG (H + L) conjugated to Alexa Fluor 647 for flow cytometry analysis. Binding efficiencies are expressed as the percent of cells positive for S1-Fc among the ACE2 expressing cells from one representative experiment with three replicates. This experiment was independently repeated three times with similar results.

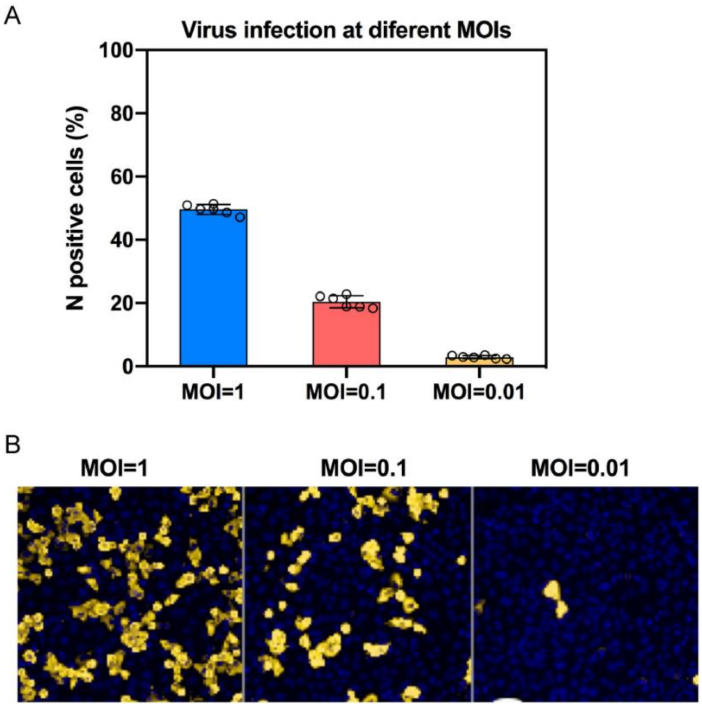


**Supplemental Figure 6**. **Correlation of N protein expression and virus infection.** A549-ACE2 cells generated by transduction of lentivector expressing the human ACE2 gene. The A549-ACE2 cells were inoculated with SARS-CoV-2 virus at the indicated moi. After 36h, the cells were fixed and immunofluorescence assay was performed to detect N protein expression. (***A***) The percentage of N protein positive cells was analyzed by Operetta High Content Imaging System (PerkinElmer). Each dot represents a sample. Error bars represent the SD of the mean from one representative experiment with six biological replicate samples. This experiment was repeated two times with similar results. (***B***) One representative image of N protein immunofluorescence from each sample was shown. N protein was stained with Alexa Fluor 555 (yellow), nuclei were stained with DAPI (blue).

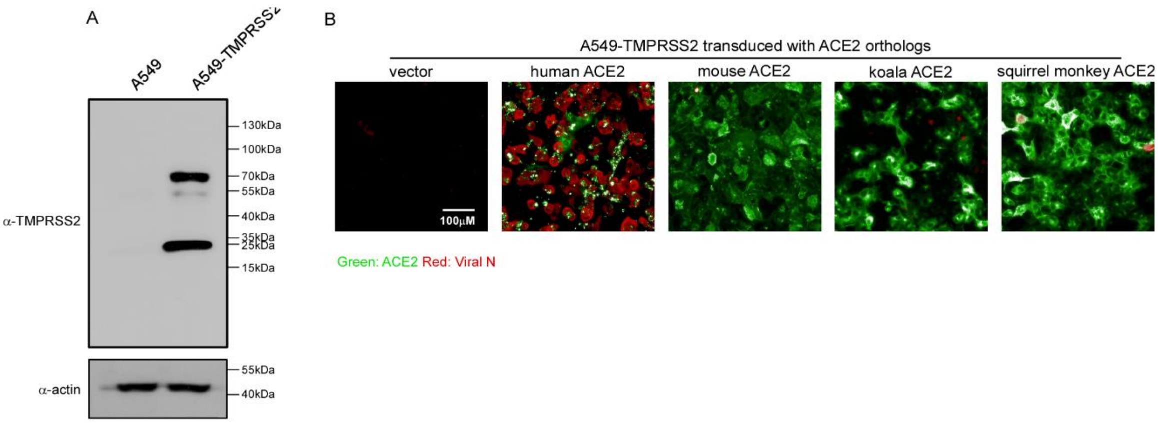


**Supplemental Figure 7**. **Assessment of New World monkey and koala ACE2 function in A549 cells expressing human TMPRSS2.** (***A***) A549 cells transduced with lentivirus expressing human TMPRSS2 was analyzed by western blotting assay to detect human TMPRSS2 expression. (***B***) A549-TMPRSS2 cells were further transduced with ACE2 orthologs as indicated, and cells were infected with SARS-CoV-2 (MOI=1). The infection was determined as described in Fig.3. This experiment was repeated twice with similar result and the representative images were shown.

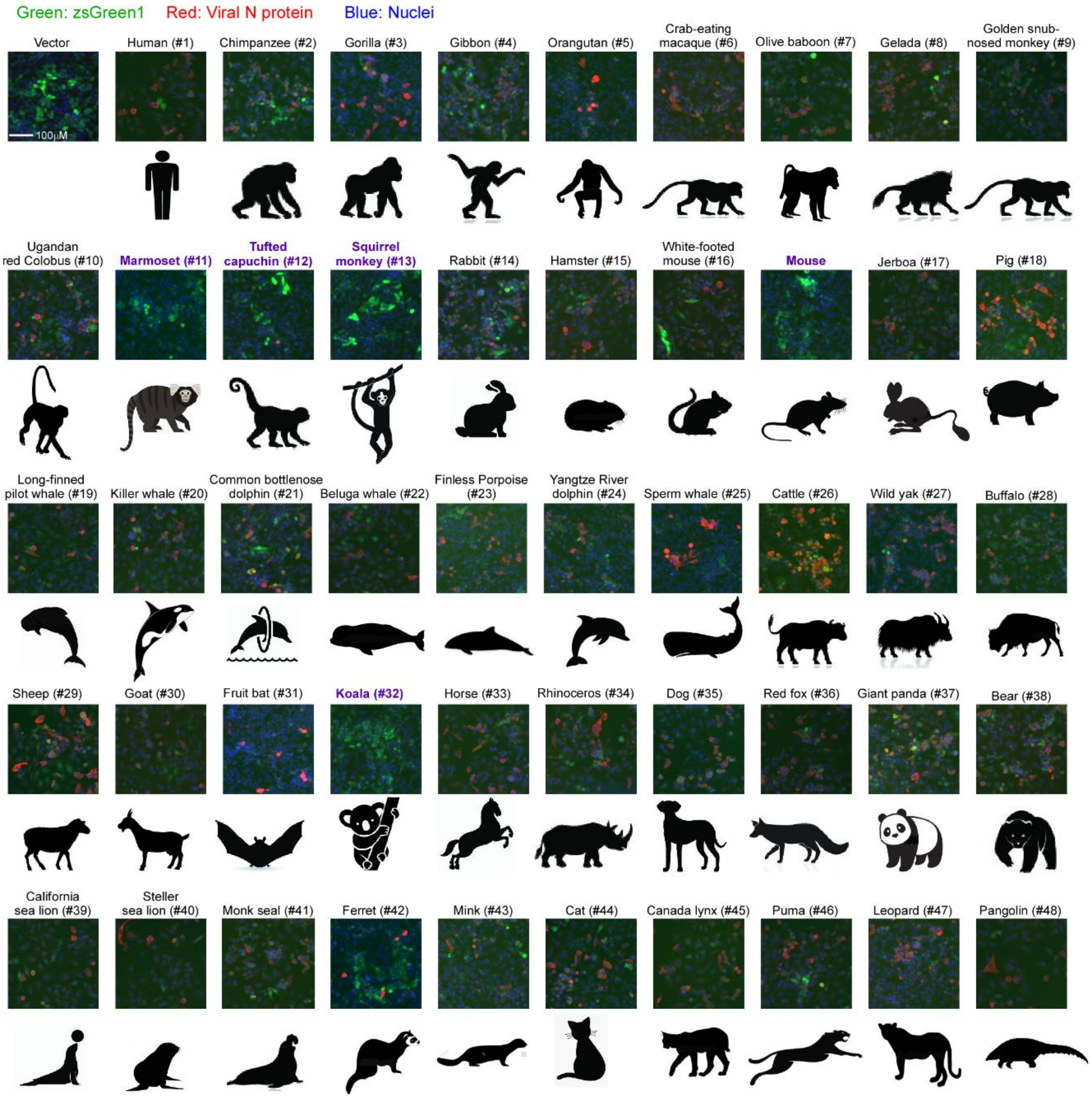


**Supplemental Figure 8**. **Functional assessment of ACE2 orthologs mediating SARS-CoV-2 virus entry.** HeLa cells transduced with lentiviruses expressing ACE2 orthologs or empty vector were infected with SARS-CoV-2 virus (MOI=1). Expression of the viral nucleocapsid protein was visualized by immunofluorescence microscopy. Viral nucleocapsid (N) protein (red) and nuclei (blue) are shown. Green signal indicates the transduction efficiency of ACE2 orthologs. Marmoset (#11), tufted capuchin (#12), squirrel monkey (#13), mouse and koala (#32) were non-permissive to SARS-CoV-2 infection, highlighted in purple. This experiment was repeated three times with similar result and the representative images were shown. The images were merged and edited using Image J software.

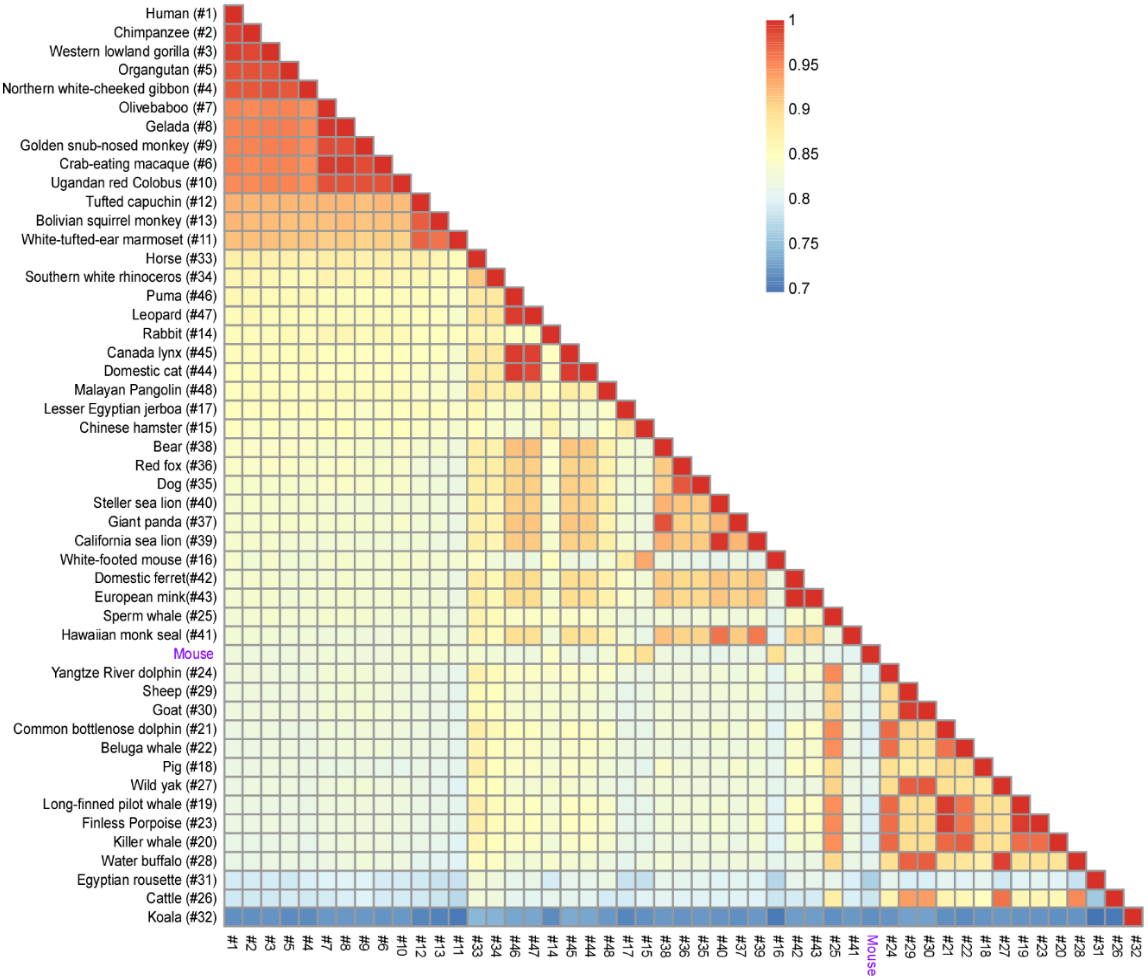


**Supplemental Figure 9**. **Protein sequence identity matrices of ACE2 from the tested species.** The ACE2 sequences from different species were analyzed using SIAS (Sequence Identity And Similarity) tool (http://imed.med.ucm.es/Tools/sias.html) to determine the percent identity of ACE2 proteins across different species. Mouse ACE2 is highlighted as red.

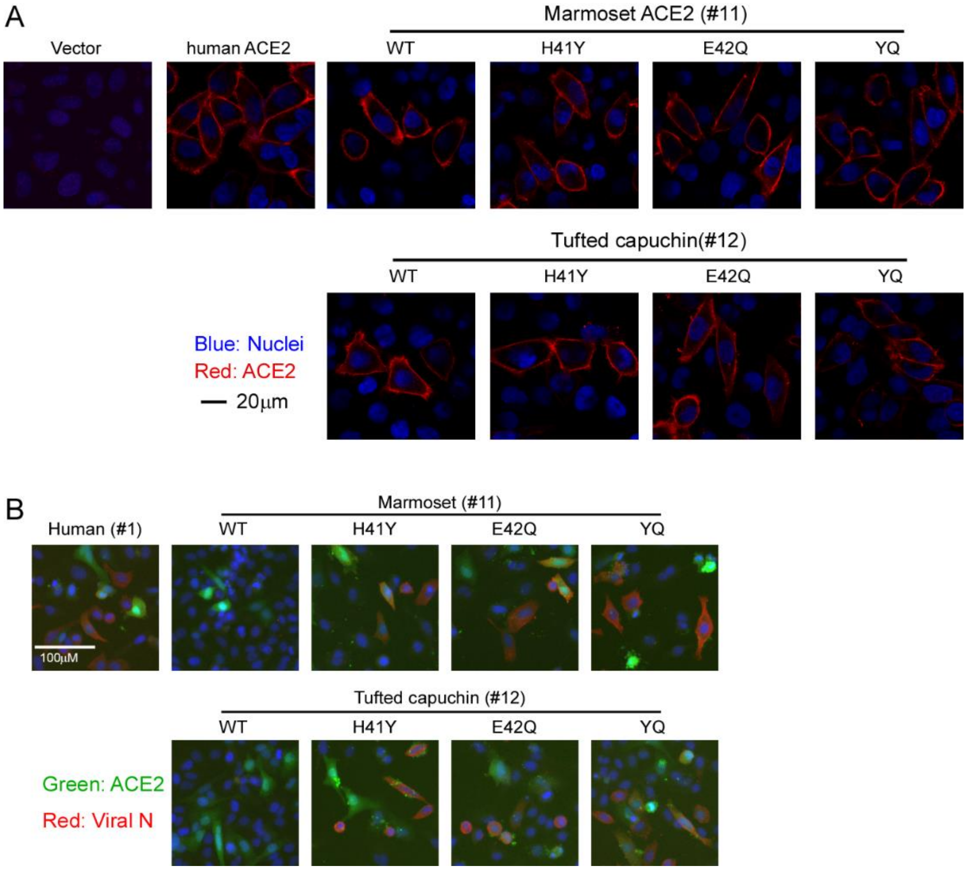


**Supplemental Figure 10**. **Cell surface localization and the receptor activities of New world monkey ACE2 orthologs or their humanized variants.** (***A***) A549 cells transduced with lentiviruses (pLVX-IRES-zsGreen) expressing ACE2 orthologs from different species and humanized variants were incubated with rabbit polyclonal antibody (Sino Biological Inc. China, Cat: 10108-T24) against ACE2. The cells were washed and then stained with 2μg/mL goat anti-rabbit IgG (H+L) conjugated with Alexa Fluor 568 and DAPI (1μg/ml). The cell images were captured with a Zeiss LSM 880 Confocal Microscope. ACE2 on cell surface was shown in the merge images processed by ZEN3.2 software. This experiment was independently repeated twice with similar result and the representative images were shown. (***B***) HeLa cells transduced with ACE2 orthologs of the indicated species or mutants were infected with SARS-CoV-2 virus (MOI=1). Expression of the viral nucleocapsid protein was visualized by immunofluorescence microscopy. Viral nucleocapsid (N) protein (red) and nuclei (blue) are shown. Green signal indicates the transduction efficiency of ACE2 orthologs. This experiment was repeated three times with similar result and the representative images were shown. The images were merged and edited using Image J software.

**SI References**


1. R. Zhang *et al.*, Mxra8 is a receptor for multiple arthritogenic alphaviruses. *Nature* **557**, 570-574 (2018).
2. J. Lan *et al.*, Structure of the SARS-CoV-2 spike receptor-binding domain bound to the ACE2 receptor. *Nature* **581**, 215-220 (2020).
